# Effect of the Terminal Acceptor Unit on the Performance of Non-Fullerene Indacenodithiophene Acceptors in Organic Solar Cells

**DOI:** 10.3390/molecules27041229

**Published:** 2022-02-11

**Authors:** Natalia Terenti, Gavril-Ionel Giurgi, Lorant Szolga, Ioan Stroia, Anamaria Terec, Ion Grosu, Andreea Petronela Crișan

**Affiliations:** 1 Department of Chemistry and SOOMCC, Faculty of Chemistry and Chemical Engineering, Babeș-Bolyai University, Cluj-Napoca, 11 Arany Janos Str., 400028 Cluj-Napoca, Romania; natalia.terenti@ubbcluj.ro (N.T.); gavril.giurgi@ubbcluj.ro (G.-I.G.); ioan.stroia@stud.ubbcluj.ro (I.S.); anamaria.terec@ubbcluj.ro (A.T.); ion.grosu@ubbcluj.ro (I.G.); 2 Optoelectronics Group, Base of Electronics Department, ETTI, Technical University of Cluj-Napoca, 28 Memorandumului Str., 400114 Cluj-Napoca, Romania; lorant.szolga@bel.utcluj.ro

**Keywords:** indacenodithiophene, electron acceptor, non-fullerene organic solar cells, structure–property relationship

## Abstract

Four acceptor–donor–acceptor (A–D–A)-type molecules bearing indacenodithiophene as donating central core and various end-capping acceptor units have been designed and synthesised as *n*-type materials suitable for organic solar cells (OSCs). The studied optical and electrochemical properties supported by theoretical calculations revealed that the nature and the strength of the terminal groups exert a decisive influence on the polymer bulk-heterojunction OSC performance.

## 1. Introduction

The ever-increasing interest in renewable energy sources has resulted in research efforts on developing photovoltaic technologies based on organic semiconductor materials as one of the most promising ultra-low-cost energy sources [1,2,3]. Furthermore, some of their inherent properties, such as flexibility, lightness, transparency, and indoor efficiency, can become an advantage in integrating organic solar cells in buildings or wearable devices [4,5]. Among the organic photovoltaics (OPVs), bulk-heterojunction (BHJ) organic solar cells (OSCs) consisting of blended electron donors and acceptors as photoactive layer have garnered a lot of attention [6,7].

For a lengthy period, fullerene and its soluble derivatives (e.g., PC_61_BM, PC_71_BM, ICBA, and others) were the dominant OSC acceptors, reaching outstanding progress with device efficiency up to 11.8% [8]. However, the drawbacks of fullerene-type electron acceptors limit their performance improvement, making the synthesis of non-fullerene acceptors (NFAs) a continuous challenge for researchers. NFAs, including polymer and small molecular non-fullerene acceptors, have gained considerable attention, mainly due to their straightforward synthesis and purification, ease of energy-levels modulation, and absorption properties, along with their morphological stability, all of which are important advantages over their conventional fullerene counterparts.

To date, a wide variety of NFA materials, including perylene diimide (PDI), naphthalene diimide (NDI), diketopyrrolopyrrole (DPP), indacenodithiophene (IDT) and benzothiadiazole (Y6) based small molecular acceptors (SMAs), that exhibit improved power conversion efficiency (*PCE*) over the fullerene materials have been developed [9]. A champion device efficiency narrowing 18% *PCE* has been achieved in a single junction device using a Y6 derivative [10]. Among the multitude of investigated NFA materials, the acceptor–donor–acceptor (A–D–A) type based on an IDT core has gradually emerged as an efficient material, contributing to a remarkable enhancement of the efficiency of BHJs up to 13% [11] (a broad list of small molecular acceptors along with their photovoltaic properties appears in a very recent review [9]). 

The optoelectronic and photovoltaic properties of such structures can be modulated by modifying the A–D–A backbone system either via chemical modifications of the donor unit (D) or by changing the strength of the electron-deficient moieties (A). Concerning the D unit, the indacenodithiophene (IDT) moiety with a coplanar structure of the five fused rings has been frequently used, due to the easily adjusted properties, such as absorption spectra, electronic energy levels, carrier transport abilities, and molecular assembling behavior [12,13,14]. Among various electron-withdrawing groups, the most explored in A–D–A photovoltaic materials are dicyanovinylene [15,16], 1,3-indanedione and its derivatives [17,18,19,20], thiazolone groups [21,22,23], etc., as A–D–A-type NFAs based on these units usually exhibit intense and broad intramolecular charge transfer (ICT) bands that can be readily expanded to the near-infrared (NIR) and hence considerably increase photocurrent generation and eventually allow for the design of semi-transparent devices. 

**Chart 1:**
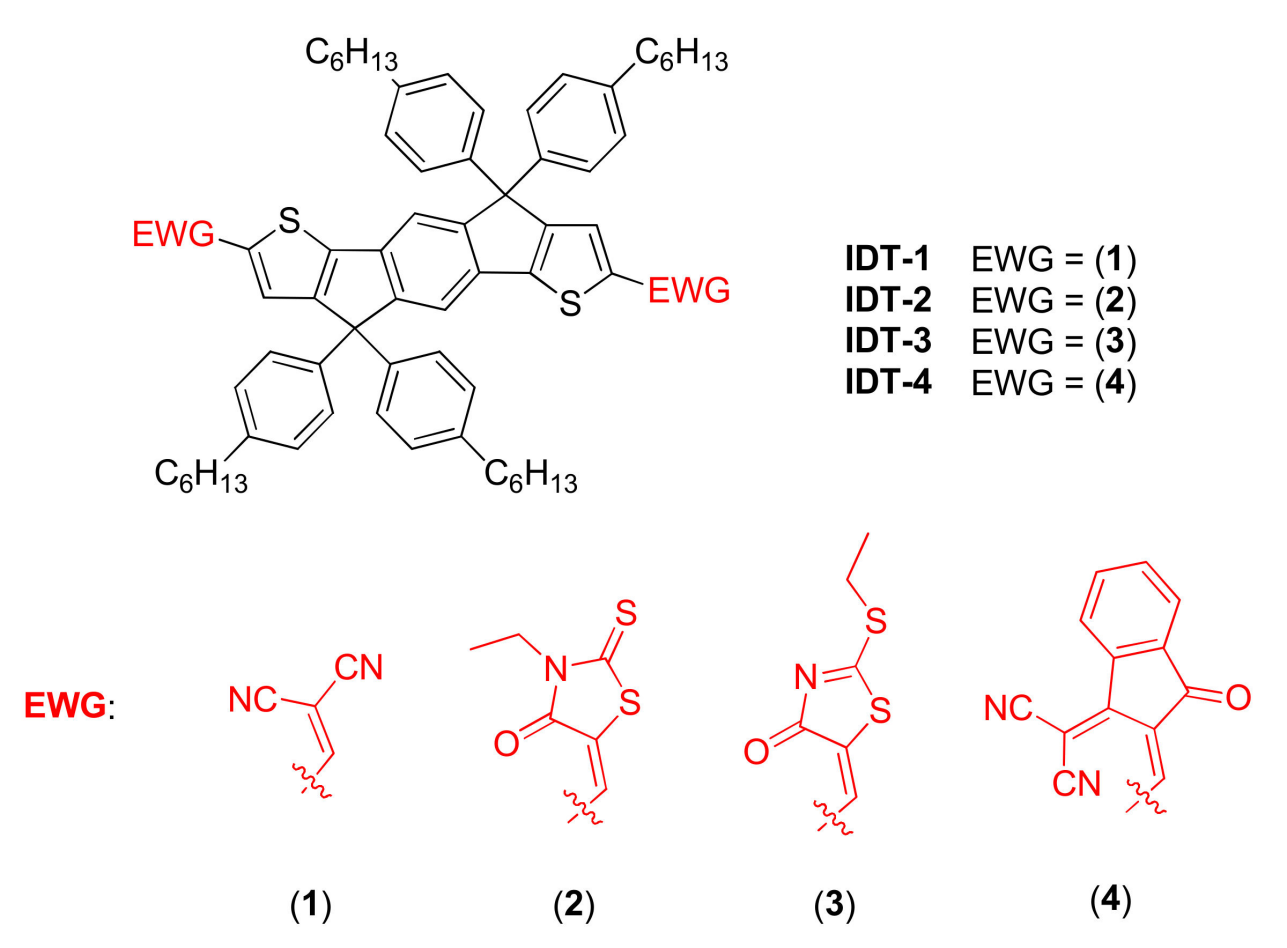
Chemical structures of target compounds **IDT-1**–**IDT-4**.

Recently, we reported the synthesis and optoelectronic properties of three A–D–A-type NFAs based on the IDT platform and the dicyanovinylene terminal groups, corresponding to three different ways of connecting the thienyl rings to the central phenyl unit of the IDT block [24]. With these results in hand, our next goal was to improve the optoelectronic and photovoltaic properties by modulating the terminal groups. We report the synthesis and comparative studies of four A–D–A-type non-fullerene acceptors (**IDT-1–IDT-4**) based on indacenodithiophene (IDT) as donor building block and various terminal electron-withdrawing groups (EWG) resulting from malononitrile (**1**), 3-ethyl-2-thioxothiazolidin-4-one (**2**), 2-(ethylthio)thiazol-4(5*H*)-one (**3**), as well as 2-(3-oxo-2,3-dihydro-1*H*-inden-1-ylidene)malononitrile (**4**) (Chart 1). The structure–property relationships concerning the impact of the EWG groups attached to the IDT core on the photovoltaic properties were detailed by testing our compounds in inverted bulk-heterojunction organic solar cells (OSCs) using the well-known P3HT as donor polymer. The results revealed that the most electron-deficient unit (**4**) gave the best photovoltaic characteristics; furthermore, special attention should be paid to impurification with the S-ethyl isomer in the rhodanine derivative that leads to a notable decrease in photovoltaic performance.

## 2. Results

### 2.1. Synthesis

**Scheme 1:**
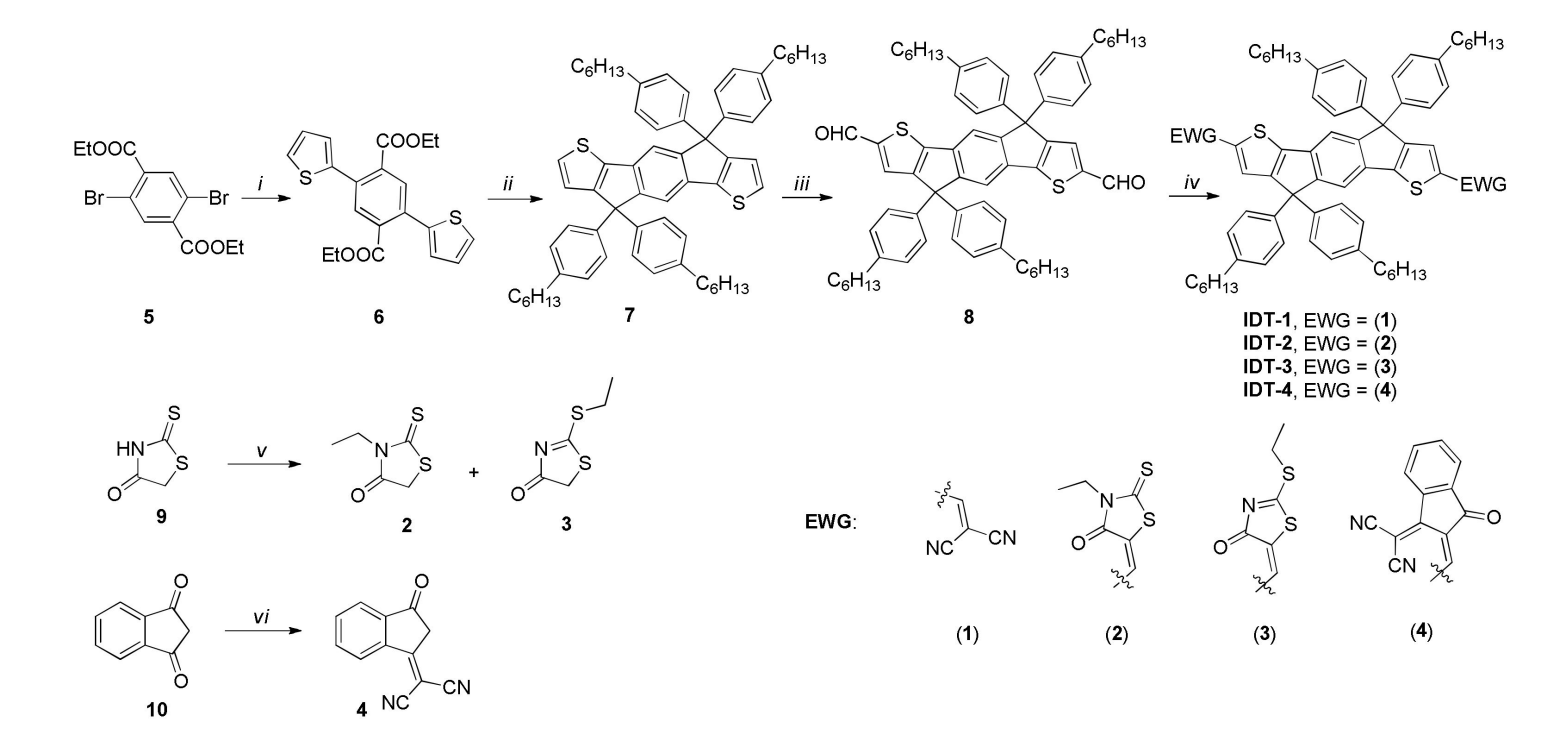
Procedure and conditions for the synthesis of A–D–A-type non-fullerene acceptors **IDT-1–IDT-4** and electron-withdrawing moieties **2–4**: (i.) Pd(PPh_3_)_4_, 2-(tributylstannyl)thiophene, toluene, 100 °C, 24 h, 79%; (ii.) *n*-BuLi, 1-bromo-4-hexylbenzene, THF, −78 °C, 15 h, rt; CH_3_COOH, H2SO_4_, reflux, 4 h, 45%; (iii.) C_2_H_4_Cl_2_, DMF, POCl_3_, 65 °C, 12 h, 56%; (iv.) for **IDT-1**: malononitrile, pyridine, toluene, 65 °C, 5 h, 74%; for **IDT-2**: 3-ethyl-2-thioxothiazolidin-4-one **2**, pyridine, toluene, 65 °C, 18 h, 57%; for **IDT-3**: 2-(ethylthio)thiazol-4(5H)-one **3**, pyridine, toluene, 65 °C, 20 h, 50%; for **IDT-4**: 2-(3-oxo-2,3-dihydro-1H-inden-1-ylidene)malononitrile **4**, pyridine, toluene, 65 °C, 8 h, 72%; (v.) CH_3_CH_2_Br, KOH, ethanol, 80 °C, overnight, 48% (**2**), 26% (**3**); vi. malononitrile, NaOAc, EtOH, 40 min, 85%.

Scheme 1 reveals the synthetic procedure of the target compounds. **IDT-1** [24] and **IDT-4** [25] were already reported in the literature. The IDT building block was prepared using a previously described multi-step synthetic protocol [24]. Firstly, 2,5-di(thiophen-2-yl) terephthalate **6** was obtained in 79% yield by reacting diethyl 2,5-dibromoterephthalate **5** with tributyl(thiophen-2-yl)stannane. A double intramolecular cyclization reaction of compound **6** with the corresponding lithiated derivative of 1-bromo-4-hexylbenzene gave the IDT core **7** in moderate yield (45%). A Vilsmeier formylation using an excess of Vilsmeier reagent afforded the central IDT building block **8** decorated with CHO groups. The target compounds **IDT-1–IDT-4** were obtained by a Knoevenagel condensation reaction of the IDT-CHO compound **8** and the corresponding electron-withdrawing moieties (malononitrile, 3-ethyl-2-thioxothiazolidin-4-one **2**, 2-(ethylthio)thiazol-4(5*H*)-one **3** and 1,1-dicyanomethylene-3-indanone **4**) in 50–74% yields. 

In an attempt to *N*-alkylate the rhodanine **9** using ethyl bromide and KOH in ethanol [26], we obtained two products, the *N*-alkylated compound **2** along with the *S*-alkylated one **3** (Scheme 1). Obtaining these two acceptor moieties, we envisaged to study the contribution of minute modifications in the acceptor moiety of *n*-type semiconductor materials in addition to the influence of various strongly electron-accepting moieties on the photovoltaic parameters. The 1,1-dicyanomethylene-3-indanone **4** was synthesized by using a reported procedure [27]. The molecular structure and purity of all compounds were proved by NMR spectroscopy and mass spectrometry. All the obtained derivatives showed high solubility in the widely-used organic solvents (e.g., dichloromethane, chloroform, *o*-chlorobenzene), facilitating their use in solution-processed bulk-heterojunction cells. 

### 2.2. Theoretical Calculations

**Figure 1:**
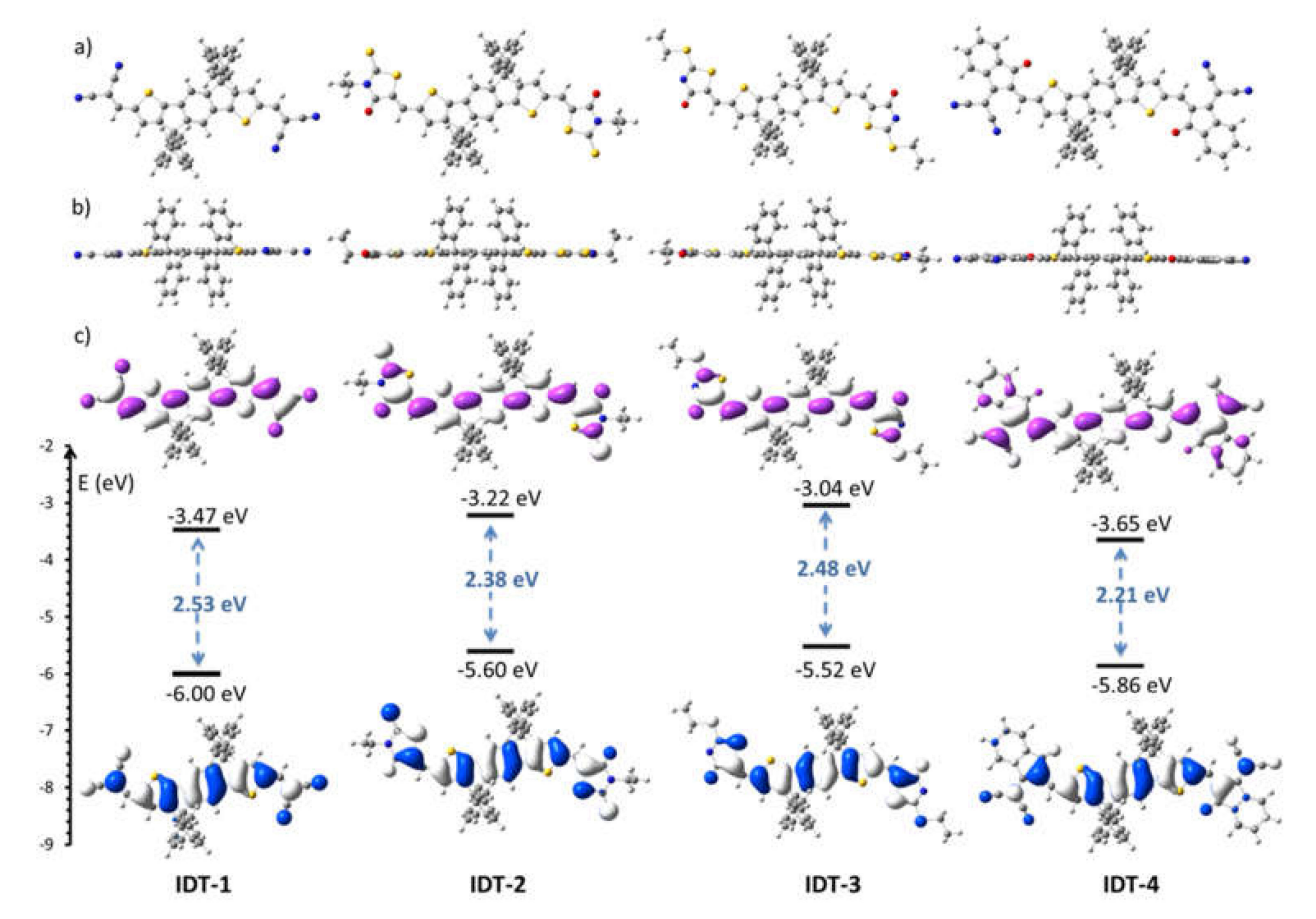
Optimized molecular geometries: top view (**a**), and lateral view (**b**), and distributions of the HOMO/LUMO canonical orbitals and their determined energies (**c**) for acceptor molecules **IDT-1**–**IDT-4** (hexyl groups were omitted for clarity).

To gain an accurate understanding of the molecular level impact of the terminal groups on the relationship between structure and properties in the previously obtained A–D–A materials, density functional theory (DFT) calculations were carried out utilizing Gaussian 09 package [28]. Becke’s three-parameter gradient-corrected functional (B3LYP-D3) with a 6-311G (d,p) basis [29,30] was used to refine the geometry and to analyze their ground states. To facilitate the calculations, the hexyl tethers were omitted. All geometries were fully optimized in dichloromethane as solvent (using the default SCRF method) without symmetry coercions and corresponding to minima. The optimized geometries of the four molecules **IDT-1–IDT-4** and the electron distribution of the HOMOs and LUMOs are shown in Figure 1. The central IDT and the terminal-accepting moieties exhibit a highly coplanar configuration, while the phenylene substituents on the IDT are situated in planes that are almost orthogonal to the backbone plane. The fully planar geometry of the main part favors -electron delocalization, which can result in improved charge mobility. Moreover, the *n*-hexylphenyl groups on the IDT central core can be expected to hinder the molecular aggregation resulting from the high degree of planarity of the molecules [31]. The HOMO orbital is essentially distributed on the donor unit in all compounds except for compound **IDT-1**, in which the HOMO wave functions are delocalized across the entire molecule. In contrast, their LUMO orbital is distributed along with the donor and acceptor units. 

The calculated energy levels of the frontier orbitals both for A–D–A systems and for individual indacenodithiophene (IDT) central moiety and different electron-withdrawing groups (EWGs) clearly confirm that the nature of the electron-accepting moiety exerts an influence on the energy gap, the data being summarized in Figure 1c, Table 1, and Chart S1. The computed results show that the EWG groups contribute only to a small decrease in the HOMO level, by maximum 0.55 eV relative to IDT, while the LUMO levels are significantly decreased in A–D–A derivatives, relative to IDT. All these data prove that the decrease in the energy gap is a direct consequence of a decrease in the LUMO levels associated with the various acceptor units. The energy level of the frontier orbitals changes distinctly with the strength of the acceptor unit. The tendency of the calculated HOMO–LUMO bandgap unfluctuates when compared to the experimentally determined optical and electrochemical bandgaps (Table 1).

### 2.3. Electrochemical and Optical Properties

The electronic characteristics of compounds **IDT-1–IDT-4** were investigated by absorption spectroscopy (in dichloromethane solution and as glass spun-casted thin films from chloroform solutions) and cyclic voltammetry (in dichloromethane in the presence of 0.1 M tetrabutylammonium hexafluorophosphate as supporting electrolyte), respectively. The appropriate parameters are summarized in Table 1.

**Figure 2:**
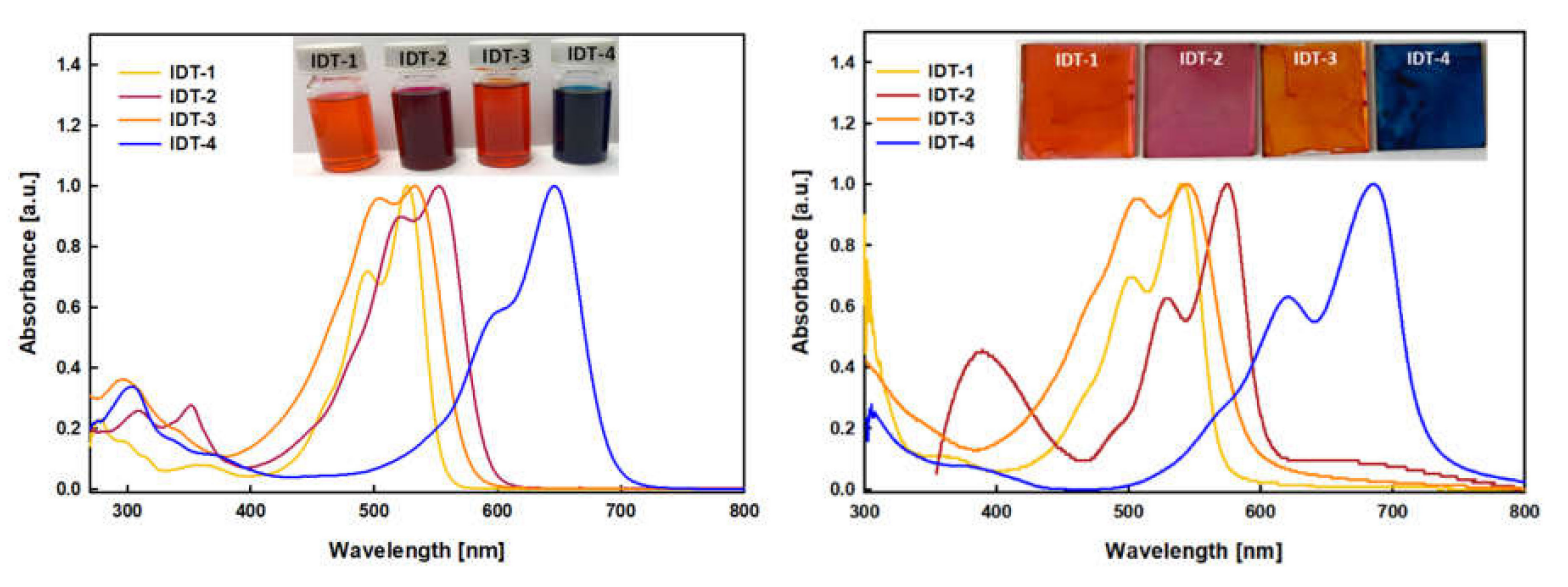
Normalized UV–vis absorption spectra of **IDT-1**–**IDT-4** in dichloromethane solution (**left**) and in thin films (**right**).

The UV–vis absorption spectra of all derivatives in dichloromethane solution (Figure 2) reveal a first band in the 270–400 nm wavelength range that can be ascribed to the π-π* transition of the IDT unit, followed by two more intense bands in the 495–645 nm region related to an internal charge transfer (ICT) taking place from the IDT donating unit to the different accepting groups. Due to the rigidity of the IDT core, all derivatives display in the low-energy region distinct shoulder peaks prior to the sharp peak due to vibronic transitions (0-0 and 0-1) as a result of their intrinsic optical properties [31]. This behavior is observed both in solution and in thin films, the optical spectra exhibiting similar absorption profiles. The data in Table 1 indicates that enhancing the electron-withdrawing ability of the terminal groups produces a bathochromic shift of the ICT band’s absorption maximum from 527 to 645 nm for **IDT-1** and **IDT-4**, respectively. By comparison, the absorption spectra of the **IDT-2** and **IDT-3** reveal that an *N*-ethylrhodanine moiety (**IDT-2**) results in a more red-shifted absorption than that of the *S*-ethylrhodanine isomer (**IDT-3**). Compared to the solutions, the absorption spectra of the thin films (Figure 2) show, as expected, a bathochromic shift of λ_max_ and a widening of the absorption band related to solid-state intermolecular interactions. The optical band gap (E_g_^opt^, Table 1) estimated from the onset of the long-wavelength thin film absorption varies from 2.13 to 1.70 eV for **IDT-1** and **IDT-4**, respectively, showing that the strength of the electron-acceptor units decreases from **IDT-4** to **IDT-1** and may influence the solid-state packing. Furthermore, these results prove that keeping the same donor unit and changing only the nature of the acceptor allows for a tuning of the band gap over *ca* 0.43 eV.

**Figure 3:**
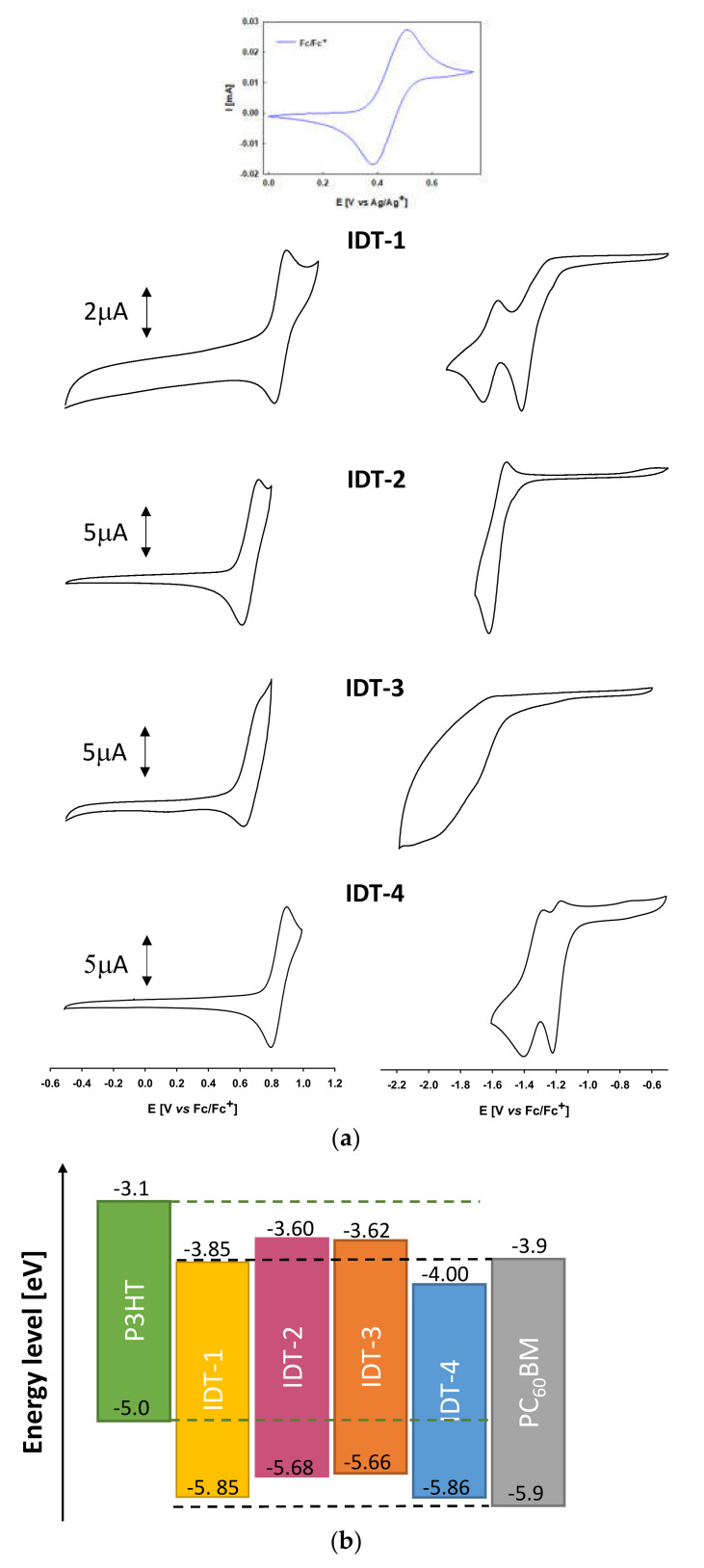
(**a**) Cyclic voltammograms of compounds **IDT-1**–**IDT-4** in 0.10 M Bu_4_NPF_6_/CH_2_Cl_2_, Pt electrodes, scan rate 100 mV s^−1^, and (**b**) energy level diagram of **IDT-1**–**IDT-4** in comparison with classical donor P3HT and fullerene acceptor PC_60_BM. The energy levels of P3HT and PC_60_BM are from [32].

Cyclic voltammograms (CV) of **IDT-1–IDT-4** (Figure 3) show a one-electron oxidation process with anodic peak potential (E_pa_) around 0.9 V for **IDT-1** and **IDT-4**, which is negatively shifted by ~ 200 mV in case of the rhodanine acceptor unit (**IDT-2** and **IDT-3**). The oxidation process is reversible for **IDT-1** and **IDT-4** and becomes quasi-reversible for **IDT-2** and irreversible for **IDT-3**. In the negative potential region, the CVs of **IDT-1** and **IDT-4** exhibit two successive reduction processes with cathodic peak potentials (E_pc_^1^ and E_pc_^2^) at −1.43 and −1.67 V for **IDT-1** shifted toward positive potentials −1.22 and −1.40 V in case of **IDT-4**. In the case of **IDT-2** and **IDT-3** the CVs display only one reduction wave with E_pc_ at −1.63 V and −1.95 V, respectively. This trend marked by the negative shift of the cathodic peak potentials confirms a decreasing ability of the end terminal groups and can also suggest a weak ICT when *S*-ethylrhodanine is used as acceptor moiety.

The frontier orbitals’ energetic levels were evaluated through the onset of the oxidation and reduction processes (Table 1). The electrochemically estimated HOMO–LUMO energy gap (E_g_^e^) along with optical data for **IDT-1** and **IDT-4** show that insertion of an indanone unit between IDT and dicyanovinylene in **IDT-4** changes the LUMO level alone, suggesting that the HOMO level is mainly related to the IDT core, while the LUMO of the compounds is sensitive to the nature of the acceptor group. The same trend is supported by DFT predictions: the electrochemical gaps are ~ 0.35–0.5 eV lower compared to the calculated HOMO–LUMO gap, which might be caused by the different nature of the processes involved in the reduction of the compounds [24]. For all synthesized compounds the LUMO levels are consistent with that of PC_60_BM derivatives, as shown in Figure 3b, suggesting their suitability for being used as *n*-type semiconductors in BHJ OSCs with P3HT.

### 2.4. Photovoltaic Properties

To further understand the structure–property interdependence of these A–D–A molecular semiconductors and to demonstrate potential applications in OSCs of all four synthesized *n*-type derivatives **IDT-1–IDT-4**, bulk-heterojunction solar cells were fabricated using P3HT as *p*-type semiconducting polymer as a result of the favorable energetic offsets. The evaluation of the photovoltaic parameters of **IDT-1–IDT-4** was carried out on inverted cells having the following architecture: indium–tin–oxide-coated glass (ITO)/zinc oxide (ZnO)/active layer (P3HT:*n*-type molecule)/molybdenum oxide (MoO_3_)/aluminum (Al). We chose to use an inverted solar architecture instead of a conventional one for its improved environmental stability [33], allowing for testing the devices under ambient conditions. Approximately 80 nm-thick active layers were obtained through the air spin-coating process from chloroform: *o*-chlorobenzene (1:1 (v:v) ratio) solutions without use of additives on a 30 nm ZnO layer, followed by thermal evaporation of a MoO_3_ film (10 nm thickness) and 100 nm of aluminum. Every ITO substrate comprises two 28 mm^2^ round cells, while a batch usually consists of six to eight cells. The device optimization processes were conducted by changing the donor/acceptor weight ratios, the details being provided in Table S5.

**Figure 4:**
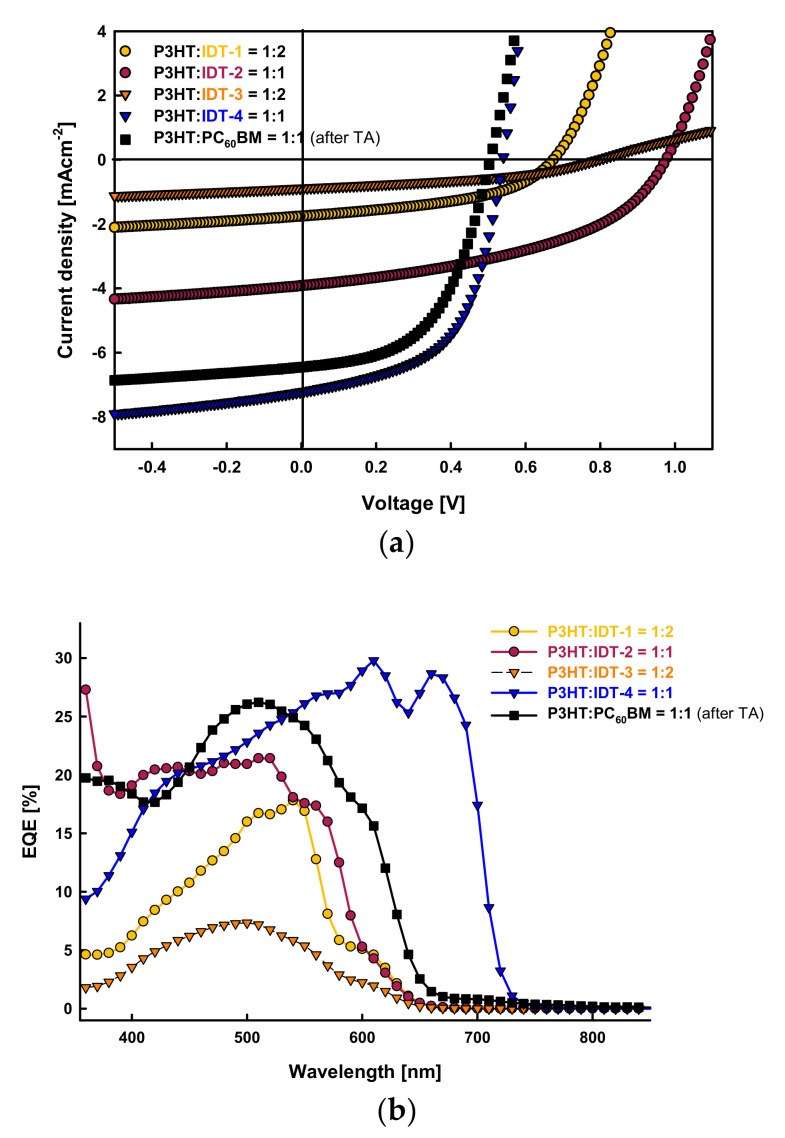
(**a**) Current density–voltage characteristics of the OSC devices in a structure of ITO/ZnO (30 nm)/photoactive layer (70–80 nm)/MoO_3_ (10 nm)/Al based on an active layer of P3HT/**IDT-1**, P3HT/**IDT-2**, P3HT/**IDT-3**, P3HT/**IDT-4**, and P3HT/**PC_60_BM**, respectively, under the illumination of AM 1.5 G, 100 mW cm^−2^; and (**b**) EQE spectra of corresponding devices.

Figure 4a,b reveal the external quantum efficiency (EQE) and the current density–voltage (J–V) characteristics of the cells based on P3HT: *n*-type molecule, recorded under AM 1.5 G simulated solar irradiation at a light power intensity of 100 mW cm^−2^, and the main photovoltaic parameters are outlined in Table 2 and Table S5.

For the P3HT/**IDT** series, in the same optimized conditions, without any thermal annealing (TA) treatment, the devices based on IDT-type NFAs, except for the cells based on **IDT-3**, yielded higher power conversion efficiency (*PCE*) relative to the fullerene acceptor PC_60_BM due to a significant improvement of the fill factor (FF) along with an increased short-circuit current density (J_sc_) value, especially in case of **IDT-2** and **IDT-4**. All devices were subjected to TA treatment, which produced no improvement of photovoltaic characteristics in the case of the P3HT/IDT series. However, there was a net enhancement of *PCE* in the case of P3HT/PC_60_BM cells owing to a simultaneous increase in the short-circuit current density (J_sc_) and fill factor (FF).

The solar cells utilizing the non-fullerene acceptor **IDT-4** gave in optimized conditions a higher *PCE* relative to the fullerene acceptor PC_60_BM due to an increased short-circuit current density (J_sc_) value of 7.23 mA cm^−2^ combined with a slight improvement of the fill factor (FF) to 56.5 %. These results could be attributed to the red-shifted absorption of P3HT/**IDT-4**, responsible for a greater photocurrent generation. The device based on P3HT/**IDT-2** delivered a *PCE* of 1.74 % with a V_oc_ of 0.97 V, J_sc_ of 3.91 mA cm^−2^, and FF of 46.3 %. The results obtained with **IDT-2** show that despite higher V_oc_ as a result of the up-shifted LUMO energy level of the **IDT-2** acceptor, the *PCE* remained low compared to the device with **IDT-4** or PC_60_BM, which may be related to the lower J_sc_ and FF attributed to the weaker light absorption from **IDT-2**. It has been shown that the V_oc_ relies on the gap between the HOMO of the donor and LUMO of the acceptor [34]; consequently, one can foresee that V_oc_ would rise as the LUMO level of the acceptor increases. Thus, for example, V_oc_ increases from 0.54 V for the cells based on **IDT-4** to 0.67 V, 0.79 V, and 0.97 V for the devices based on **IDT-1**, **IDT-3**, and **IDT-2**, respectively. Although the devices utilizing **IDT-1** and **IDT-3** as acceptor exhibited a higher V_oc_ of 0.67 V and 0.79 V, relative to **IDT-4**, the J_sc_ of these devices is four and eight times lower, respectively, in comparison with that obtained with **IDT-4**.

Among all of the studied NFAs, the absorber with the highest efficiency (red-edge onset at 725 nm—see supporting info, ~1.71 eV) is the P3HT/**IDT-4** blend that generated the best photovoltaic characteristics in the absence of additives or thermal annealing, thus simplifying the process of fabrication and enhancing the reproducibility of the device performance.

To further comprehend the device efficiency, the external quantum efficiency (EQE) responses of the OSCs based on the five acceptors were recorded under monochromatic irradiation (Figure 4b). It can be observed that all EQE spectra showed a broad photo response extending from 300 to 680 nm which agree well with their corresponding absorption profile of the blend films (Figure S18, ESI). Among all four non-fullerene acceptors, the **IDT-4**-based devices with higher *PCE* values showed the widest photo response with a maximum EQE value of 22%, which can be attributed to a higher absorption of **IDT-4** relative to the other synthesized non-fullerene acceptors, and which also confirms the contribution to the higher J_sc_ of the OSCs with **IDT-4** as acceptor.

Among the newly obtained NFAs, **IDT-2** and **IDT-3**, electron-mobility was evaluated for the **IDT-2** compound, giving better photovoltaic characteristics than **IDT-3**. The electron-mobility of the pristine **IDT-2** film was measured via the space-charge-limited current (SCLC) method by using devices with ITO/ZnO (20nm)/**IDT-2** (50 nm)/Bphen (15 nm)/Ag (100 nm) arhitecture. It was found that the **IDT-2**-based neat film possesses average electron mobility (μ_e_) of 1.27 x 10^−6^ cm^2^V^−1^s^−1^, which is lower than that of the film based on PTB7-Th:**IDT-4** (1.86 x 10^−5^ cm^2^V^−1^s^−1^) [35] with a layer two times thicker. The current-voltage (log J-log V) curve used for the extraction of μ_e_ is shown in Figure S19, SI.

### 2.5. Dipole Moment Calculations

It was shown that there is a direct correlation between the magnitude of the dipole moment change (Δµ_ge_) and photovoltaic characteristics data [36,37]. Hence, to obtain additional information regarding the effect of the end-group on the charge generation in BHJ OSC, we analyzed the dipolar changes between the ground and excited state (Δµ_ge_) using the DFT method. The calculated dipole moments for **IDT-1**–**IDT-4** compounds are listed in Table 3. It can be seen from the calculated values that all four compounds have very low dipole moments as a result of both EWG groups on the IDT unit pulling the electron density in opposite directions. However, there are subtle differences concerning the dipolar changes (Δµ_ge_) of the four NFAs, the larger Δµ_ge_ upon HOMO–LUMO transition being observed in the case of **IDT-4**, suggesting an enhanced polarized exciton that facilitates favorable charge generation and makes charge separation easier.

The key factor determining high photovoltaic performances in BHJ OSCs is the charge generation process, including exciton dissociation and charge recombination. Our results prove that the Δµ_ge_ has a strong influence on the V_oc_, J_sc_, and PCE values (Table 2 and Table 3). The J_sc_ and *PCE* values increase with the enhancement of Δµ_ge_ while the V_oc_ decreases with the enhanced polarizability of the excited state. Even if both **IDT-2** and **IDT-3** exhibit the same Δµ_ge_ values, there are significant differences in their photovoltaic performances. We reasoned that the poor performance of **IDT-3** may be the result of poor exciton dissociation and very fast charge recombination as a consequence of very low ground- and excited-state dipole moment values.

## 3. Experimental Section

### 3.1. General

Commercially available reagents and chemicals were used with no further purification. Solvents were purified and dried when needed through standard techniques. Thin-layer chromatography (TLC) was carried out using Silica gel 60 chromatography plates F_254_ and visualized by UV irradiation at 254 nm. Preparative column chromatography was conducted using analytical-grade solvents on silica gel (technical grade, pore size 60 Å). NMR spectra were measured with a Bruker AVANCE III 400 or 600 (^1^H, 400 or 600 MHz and ^13^C, 100 or 150 MHz). Chemical shifts are reported in ppm relative to TMS as an internal reference, and coupling constants (*J*) are given in Hz. High-resolution mass spectra (HRMS) in the positive ion mode, using APCI or electrospray techniques, were recorded with an LTQ XL OrbitrapThermoScientific mass spectrometer. Elemental analyses were performed using a FLASH EA 1112-Thermo Scientific elemental analyzer to establish C, H, N, and S contents. UV–vis spectra were recorded with a Cecil Super Aquarius spectrophotometer. Electrochemical measurements were carried out in dichloromethane solution with a BioLogic SP-150 potentiostat in a standard three-electrode cell using platinum working and counter electrodes and an Ag/Ag+ reference electrode in 0.1 M nBu4PF6 as supporting electrolyte, with a scan rate of 100 mVs^−1^. All potentials are reported relative to an Fc/Fc+ internal standard. Solutions were degassed by argon bubbling before each experiment. A DFT computational study was performed using the Gaussian 09 package, employing the dispersion corrected form of B3LYP exchange-correlation functional (B3LYP-D3, with D3 standing for Grimme’s dispersion corrections) and 6-311G(d,p) as basis set [28,29,30]. 

### 3.2. Synthesis of Compounds

Compounds **2**,**3** [26], **4** [27], **6**, **7**, **8**, **IDT-1** [24], and **IDT-4** [25] were obtained using known procedures.

General procedure: 0.2 mL pyridine was added in inert conditions to a solution containing derivative **8** (0.3 mmol) and the corresponding electron-withdrawing moiety **2** or **3** (0.9 mmol) in dry toluene (30 mL). The reaction mixture was stirred overnight at 65 °C and then the solvent was removed in vacuo. The crude product was chromatographed on silica gel using toluene as eluent to give the targeted derivatives **IDT-2** and **IDT-3** in 57 % and 50% yield, respectively.

5,5′-((4,4,9,9-tetrakis(4-hexylphenyl)-4,9-dihydro-s-indaceno[1,2-b:5,6-b’]dithiophene-2,7 diyl)bis(methaneylylidene))bis(3-ethyl-2-thioxothiazolidin-4-one) IDT-2: Yield: 57% (220 mg), R_f_ = 0.8 (toluene), red solid. ^1^H NMR (400 MHz, CDCl_3_), δ (ppm): 7.83 (s, 2H), 7.52 (s, 2H), 7.29 (s, 2H), 7.14–7.08 (overlapped signals, 16H), 4.15 (q, J = 7.2 Hz, 4 H), 2.56 (m, 8 H), 1.58 (m, 8 H), 1.34–1.27 (overlapped signals, 30 H), 0.87 (t, J = 6.4 Hz, 12 H) ppm. ^13^C NMR (100 MHz, CDCl_3_), δ (ppm): 191.8, 167.4, 158.1, 154.7, 149.5, 142.3, 141.4, 140.8, 135.8, 129.7, 128.8, 127.8, 126.1, 119.9, 118.6, 77.4, 63.2, 35.7, 31.8, 31.5, 29.3, 22.8, 14.3, 12.3 ppm. HRMS (APCI+): Calcd. for C_76_H_85_N_2_O_2_S_6_ [M + H]^+^: 1249.4930, found: 1249.4976. Anal. calcd for C_76_H_84_N_2_O_2_S_6_: C, 73.03; H, 6.77; N, 2.24; S, 15.39. Found: C, 72.95; H, 6.78; N, 2.23; S, 15.41.

5,5′-((4,4,9,9-tetrakis(4-hexylphenyl)-4,9-dihydro-s-indaceno[1,2-b:5,6-b’]dithiophene-2,7-diyl)bis(methaneylylidene))bis(2-(ethylthio)thiazol-4(5H)-one) IDT-3: Yield: 50 % (190 mg), R_f_ = 0.5 (toluene), orange solid. ^1^H NMR (400 MHz, CDCl_3_), δ (ppm): 7.95 (s, 2 H), 7.50 (s, 2 H), 7.28 (s, 2 H), 7.13–7.07 (overlapped signals, 16H), 3.42 (q, J = 7.6 Hz, 4 H), 2.56 (m, 8 H), 1.60 (m, 8 H), 1.48 (t, J = 7.6 Hz, 6 H), 1.35–1.25 (overlapped signals, 24 H), 0.86 (m, 12 H). ^13^C NMR (100 MHz, CDCl_3_), δ (ppm): 189.9, 180.0, 157.9, 156.3, 154.6, 147.7, 142.2, 140.8, 135.8, 128.7, 128.6, 128.5, 127.8, 123.5, 118.6, 77.3, 35.6, 31.8, 31.4, 29.8, 29.2, 22.7, 14.5, 14.2. HRMS (ESI+): Calcd for C_76_H_85_N_2_O_2_S_6_ [M + H]^+^: 1249.4930, found: 1249.4905. Anal. calcd for C_76_H_84_N_2_O_2_S_6_: C, 73.03; H, 6.77; N, 2.24; S, 15.39. Found: C, 72.99; H, 6.79; N, 2.23; S, 15.42.

2,2′-((4,4,9,9-tetrakis(4-hexylphenyl)-4,9-dihydro-s-indaceno[1,2-b:5,6-b’]dithiophene-2,7-diyl)bis(methanylylidene))dimalononitrile IDT-1. The aforementioned derivative was prepared from dialdehyde 8 and malononitrile 1 using a previously reported procedure [24], having same analytical data: ^1^H NMR (600 MHz, CDCl_3_), δ (ppm): 7.76 (s, 2 H), 7.61 (s, 2 H), 7.57 (s, 2 H), 7.08 (overlapped signals, 16 H), 2.56 (t, J = 7.8 Hz, 8 H), 1.58 (m, 8 H), 1.34–1.27 (overlapped signals, 24 H), 0.87 (m, 12 H). 

^13^C NMR (150 MHz, CDCl_3_), δ (ppm): 157.9, 156.0, 153.4, 151.1, 142.7, 140.0, 138.9, 136.2, 134.2, 128.9, 127.6, 119.8, 114.4, 113.8, 75.7, 63.2, 35.6, 31.8, 31.4, 29.2, 22.7, 14.2. HRMS (APCI+): Calcd for C_72_H_75_N_4_S_2_ [M + H]^+^: 1059.5428, found 1059.5477. Anal. calcd for C_72_H_74_N_4_S_2_: C, 81.62; H, 7.04; N, 5.29; S, 6.05. Found: C, 81.56; H, 7.03; N, 5.30; S, 6.04.

2,2′-((4,4,9,9-tetrakis(4-hexylphenyl)-4,9-dihydro-s-indaceno[1,2-b:5,6-b’]dithiophene-2,7-diyl)bis(methaneylylidene))bis(3-oxo-2,3-dihydro-1H-indene-2,1-diylidene))dimalononitrile IDT-4: This derivative was obtained from dialdehyde 8 and indandione derivative 4 following a method previously reported in literature [25], affording the same analytical data: ^1^H NMR (600 MHz, CDCl_3_), δ (ppm): 8.90 (s, 2 H), 8.69 (d, J = 7.8 Hz, 2 H), 7.91 (m, 2 H), 7.78–7.71 (overlapped signals, 8 H), 7.15–7.10 (overlapped signals, 16H), 2.57 (t, J = 7.8 Hz, 8H), 1.59 (m, 8 H), 1.35–1.27 (overlapped signals, 24H), 0.86 (m, 12 H). ^13^C NMR (150 MHz, CDCl_3_), δ (ppm): 188.6, 160.5, 158.9, 158.1, 156.5, 142.5, 141.6, 140.5, 140.1, 139.6, 138.6, 137.3, 137.1, 135.4, 134.7, 128.9, 127.8, 125.5, 124.0, 122.7, 120.1, 114.7, 114.6, 69.6, 63. 2, 35.7, 31.9, 31.5, 29.2, 22.7, 14.3. HRMS (APCI+): Calcd for C_90_H_83_N_4_O_2_S_2_ [M + H]^+^: 1315.5952, found 1315.5850. Anal. calcd for C_90_H_82_N_4_O_2_S_2_: C, 82.16; H, 6.28; N, 4.26; S, 4.87. Found: C, 81.97; H, 6.27; N, 4.27; S, 4.86.

### 3.3. Device Fabrication and Characterization

Solution-processed BHJ OSCs were assembled in an inverted device configuration (ITO/ZnO/P3HT:A–D–A/MoO_3_/Al). Indium–tin–oxide (ITO)-coated glass slides of 24 × 25 × 1.1 mm with a sheet resistance of R_S_ = 7 Ω/sq were purchased from Praezisions Glas & Optik GmbH. Following an ultrasonic cleaning with diluted Deconex^®^ 12 PA-x solution (2% in water), distilled water, ethanol, and isopropanol (each wash taking 10 min), the ITO electrodes were oven-dried at 100 °C. Once dried, the ITO plates were treated with UV-ozone (UV/Ozone ProCleaner Plus, Bioforce Nanosciences) for 20 min. A 30 nm layer of ZnO was deposited on the surface of ITO-patterned glass using a solution of zinc acetate (198 mg) and ethanolamine (0.54 mL) in ethanol (6 mL). After annealing the ZnO layer thermally at 200 °C for 15 min, the active layer was deposited through spin-coating in ambiental atmosphere over the ZnO layer from a 6 mg/mL blended solution of P3HT:A–D–A (see Table 2 and Table S5 for optimum P3HT:A–D–A ratio in each case) dissolved in chloroform: chlorobenzene (1:1, v:v). Subsequently, the completion of the devices was done by consecutive thermal evaporation processes of MoO_3_ (10 nm) and aluminum (100 nm) at a pressure of 1.5 × 10^−6^ Torr through a shadow mask defining two cells of 28 mm^2^ on each ITO substrate. The current-voltage characteristics (J-V) were measured in the dark and under illumination using a Keithley 236 source-measure unit and a home-made acquisition program. The light source is a solar simulator “AM1.5 Solar Constant 575 PV” (Steuernagel Lichttecknik, equipped with a metal halogen lamp). The light intensity was measured by a broad-band power meter (13PEM001, Melles Griot). The EQE measurements were recorded under ambient atmosphere using a spectroscopy system that includes a halogen lamp (Osram) with an Action Spectra Pro 150 monochromator, a lock-in amplifier (PerkinElmer 7225) and a S2281 photodiode (Hamamatsu).

The electron mobility of **IDT-2** film was evaluated via the space-charge-limited current (SCLC) method; the devices were fabricated with a structure of ITO/ZnO/**IDT-2**/ /Bphen/Ag. The active layer, **IDT-2**, was deposited through spin-coating in an ambiental atmosphere over the ZnO layer from a 20 mg /mL solution (chloroform: chlorobenzene (1:1, *v*:*v*)). The J-V curve of devices was fitted by using the Mott–Gurney equation: J = 9ε_o_ε_r_µV^2^/8L^3^, where J is the current density, ε_0_ is the permittivity of the vacuum, ε_r_ is the permittivity of the active layer, µ is the electron mobility, V is the internal voltage of the device, and L is the film thickness of the active layer.

## 4. Conclusions

A series of A–D–A conjugated systems that employ the same conjugated backbone, the IDT core, as a donor and different electron-acceptor groups were investigated for use in solution-processed bulk-heterojunction solar cells. Spectroscopic and electrochemical studies endorsed by theoretical calculations support the potential applicability of these derivatives as *n*-type materials in OSCs. An in-depth study of these compounds showed the impact of both the nature and the strength of the electron-withdrawing moiety on the energy levels, dipole moment, and light-harvesting properties of the molecules, and consequently the OSC efficiency of A−D−A-type materials. A first study of the results obtained in BHJ OSCs using P3HT as a donor and **IDT-1–IDT-4** as acceptors led to *PCE*s in the 0.29–2.21% range, without any additives or annealing treatment. Nonetheless, this work shows that dicyanovinylindanone **4** and *N*-ethylrhodanine **2** as electron-withdrawing groups are promising candidates for designing new *n*-type semiconductors. Interestingly, despite the similar energy levels and absorption properties of the two isomers of rhodanine, the S-ethyl rhodanine isomer **3** had a deleterious effect on the photovoltaic parameters, probably due to a poor excited-state dipole moment compared to the *N*-ethyl rhodanine isomer **2**.

## Figures and Tables

**Table 1:** The corresponding optoelectronic data of **IDT-1–IDT-4**.

	IDT-1	IDT-2	IDT-3	IDT-4
**optical**				
λ_max_^a^ [nm]	495, **527**	523, **555**	505, **533**	598 (sh), **645**
E [ev] ^b^	2.18	2.03	2.10	1.74
λ_max_^c^ [nm]	502, **540**	530, **576**	506, **544**	620, **686**
E_g_^opt^ [ev] ^d^	2.13	2.04	2.10	1.70
**electrochemical**				
*E*_pa_ [V]	0.89	0.72	0.72	0.90
*E*_pc_^1^, E_pc_^2^ [V]	−1.43, −1.67	−1.63	−1.95	−1.22, −1.40
*E*_HOMO_ [eV] ^e^	−5.85	−5.68	−5.66	−5.86
*E*_LUMO_ [eV] ^e^	−3.85	−3.60	−3.62	−4.00
E_g_ [eV] ^e^	2.00	2.08	2.04	1.86
**B3LYP-D3/6-311G(d,p)**				
*E*_HOMO_ [eV] ^f^	−6.00	−5.60	−5.52	−5.86
*E*_LUMO_ [eV] ^f^	−3.47	−3.22	−3.04	−3.65
E_g_ [eV]	2.53	2.38	2.48	2.21

^a^ In CH_2_Cl_2_ solution. ^b^ From the long-wavelength absorption edge in solution. ^c^ Thin films spun-cast on glass. ^d^ From the maximum absorption onset in thin films. ^e^ From the onset of oxidation and reduction processes using the equations E_HOMO_ = −(E_ox_ + 5.1) eV and E_LUMO_ = −(E_red_ + 5.1) eV. All potentials are referenced to the Fc/Fc^+^ couple as internal standard. ^f^ Values calculated by DFT.

**Table 2:** Parameters of BHJ OSCs based on P3HT:**IDT-1**–**IDT-4** and P3HT:**PC_60_BM** measured at 100 mWcm^−2^ AM 1.5 G illumination (data in italics represent the average between 6–8 cells; data in bold are the top results for each series).

*n*-Type Molecule	P3HT/*n*-Type Molecule Ratio (wt %)	Active Layer Thickness (nm)	Annealing T (°C)	V_oc_ (V)	J_sc_ (mAcm−^2^)	FF (%)	PCE (%)
**IDT-1**	1:2	70	**-**	**0.67** (*0.65*)	**1.76** (*1.71*)	**46.32** (*46.71*)	**0.55** (0.52)
**IDT-2**	1:1	80	**-**	**0.97** (*0.95*)	**3.91** (*3.79*)	**45.56** (*45.00*)	**1.74** (*1.67*)
**IDT-3**	1:2	60	**-**	**0.79** (*0.75*)	**0.89** (*0.85*)	**41.31** (*38.75*)	**0.29** (*0.26*)
**IDT-4**	1:1	80	**-**	**0.54** (*0.53*)	**7.23** (*7.15*)	**56.53** (*56.04*)	**2.21** (*2.16*)
**PC_60_BM**	1:1	80	**-**	**0.58** (*0.57*)	**3.32** (*2.77*)	**17.45** (*15.56*)	**0.34** (*0.26*)
**PC_60_BM**	1:1	80	130	**0.51**(*0.49*)	**6.44** (*6.05*)	**52.98** (*50.30*)	**1.72** (*1.51*)

**Table 3:** Calculated dipole moments for **IDT-1**–**IDT-4** compounds.

Compound	µ_g_ (D)	µ_e_ (D)	Δµ_ge_ (D)
**IDT-1**	0.16	0.17	0.02
**IDT-2**	0.40	0.43	0.03
**IDT-3**	0.01	0.03	0.03
**IDT-4**	0.39	0.45	0.06

µ_g_ = ground-state dipole moment; µ_e_ = excited-state dipole moment; Δµ_ge_ = difference between ground- and excited-state dipole moment, calculated as: Δµ_ge_ = [(µ_gx_ − µ_ex_)^2^ + (µ_gy_ − µ_ey_)^2^ + (µ_gz_ − µ_ez_)^2^]^1/2^.

## Supplementary Materials

The following are available online. Figure S1: ^1^H NMR (CDCl_3_, 600 MHz) spectrum of compound IDT-1, Figure S2: ^13^C NMR (CDCl_3_, 150 MHz) spectrum of compound **IDT-1**, Figure S3: APCI (+)-HRMS spectrum of compound IDT-1, Figure S4: ^1^H NMR (CDCl_3_, 400 MHz) spectrum of compound IDT-2, Figure S5: ^13^C NMR (CDCl_3_, 100 MHz) spectrum of compound IDT-2, Figure S6: APCI (+)-HRMS spectrum of compound IDT-2, Figure S7: ^1^H NMR (CDCl_3_, 400 MHz) spectrum of compound IDT-3, Figure S8: ^13^C-APT NMR (CDCl_3_, 100 MHz) spectrum of compound IDT-3, Figure S9: ESI (+)-HRMS spectrum of compound IDT-3, Figure S10: ^1^H NMR (CDCl_3_, 600 MHz) spectrum of compound IDT-4, Figure S11: ^13^C-APT NMR (CDCl_3_, 150 MHz) spectrum of compound IDT-4, Figure S12: APCI (+)-HRMS spectrum of compound IDT-4, Figure S13: DFT calculated structure of IDT-1, Figure S14: DFT calculated structure of IDT-2, Figure S15: DFT calculated structure of IDT-3, Figure S16: DFT calculated structure of IDT-4, Figure S17: J-V characteristics of BHJ OSCs for different P3HT: *n*-type molecule ratios. (a) IDT-1, (b) IDT-2, (c) IDT-3 and (d) IDT-4, Figure S18: Absorption spectra of P3HT: *n*-type molecule blend films, Figure S19: Log J-log V curve of the electron-only devices. Table S1: DFT coordinates of IDT-1, Table S2: DFT coordinates of IDT-2, Table S3: DFT coordinates of IDT-3, Table S4: DFT coordinates of IDT-4, Table S5: Photovoltaic characteristics of IDT-1, IDT-2, IDT-3, IDT-4 and PC_60_BM under various conditions. Chart S1: HOMO-LUMO energy levels for the IDT core and electron-withdrawing groups.
