# Supplementary material for: Effect of the Terminal Acceptor Unit on the Performance of Non-Fullerene Indacenodithiophene Acceptors in Organic Solar Cells
Source: Molecules. 2022 Feb 11;27(4):1229. doi: 10.3390/molecules27041229 (PMC8877381; doi:10.3390/molecules27041229)
Supplement: Supplementary file 1 [file molecules-27-01229-s001.zip › molecules-1547804-supplementary.pdf]

Supporting Information

for

Effect of the terminal acceptor unit on the performance of non-fullerene indacenodithiophene acceptors in organic solar cells

by

Natalia Terenti,[1§] Gavril-Ionel Giurgi,[1§,2] Lorant Szolga,[1,2] Ioan Stroia,[1] Anamaria Terec,[1] Ion Grosu[1] and Andreea Petronela Crișan*[1]

[1] Department of Chemistry and SOOMCC, Faculty of Chemistry and Chemical Engineering, Babes-Bolyai University, Cluj-Napoca, Romania. E-mail: andreea.crisan@ubbcluj.ro
[2] Optoelectronics Group, Base of Electronics Department, ETTI, Technical University of Cluj-Napoca, Cluj-Napoca, Romania.
§ : these two authors contributed equally to the work

Table of Contents

Figure S1. ^{1}H NMR ($CDCl_3$, 600 MHz) spectrum of compound IDT-1 .... **S3**

Figure S2. ^{13}C NMR ($CDCl_3$, 150 MHz) spectrum of compound IDT-1 .... **S3**

Figure S3. APCI (+)-HRMS spectrum of compound IDT-1 ....**S4**

Figure S4. ^{1}H NMR ($CDCl_3$, 400 MHz) spectrum of compound IDT-2.................................... **S4**

Figure S5. ^{13}C NMR ($CDCl_3$, 100 MHz) spectrum of compound IDT-2 ....**S5**

Figure S6. APCI (+)-HRMS spectrum of compound IDT-2 ....**S5**

Figure S7. ^{1}H NMR ($CDCl_3$, 400 MHz) spectrum of compound IDT-3 ....**S6**

Figure S8. ^{13}C-APT NMR ($CDCl_3$, 100 MHz) spectrum of compound IDT-3 ....**S6**

Figure S9. ESI (+)-HRMS spectrum of compound IDT-3 ....**S7**

Figure S10. ^{1}H NMR ($CDCl_3$, 600 MHz) spectrum of compound IDT-4 ....**S7**

Figure S11. ^{13}C-APT NMR ($CDCl_3$, 150 MHz) spectrum of compound IDT-4 ....**S8**

Figure S12. APCI (+)-HRMS spectrum of compound IDT-4 ....**S8**

Figure S13. DFT calculated structure of IDT-1 ....**S9**

Figure S14. DFT calculated structure of IDT-2 ....**S9**

Figure S15. DFT calculated structure of IDT-3...**S9**

Figure S16. DFT calculated structure of IDT-4 ....**S10**

Table S1. DFT coordinates of IDT-1 ....**S10**

Table S2. DFT coordinates of IDT-2 ....**S12**

Table S3. DFT coordinates of IDT-3 ....**S15**

Table S4. DFT coordinates of IDT-4 ....**S17**

Figure S17. *J-V* characteristics of BHJ OSCs for different P3HT : *n*-type molecule ratios. (a) IDT-1, (b) IDT-2, (c) IDT-3 and (d) IDT-4 ....**S21**

Figure S18. Absorption spectra of P3HT : *n*-type molecule blend films **S21**

Figure S19. Log J-log V curve of the electron-only devices...**S22**

Table S5. Photovoltaic characteristics of IDT-1, IDT-2, IDT-3, IDT-4 and $PC_{60}BM$ under various conditions **S23**

Chart S1. HOMO-LUMO energy levels for the IDT core and electron-withdrawing groups...**S24**

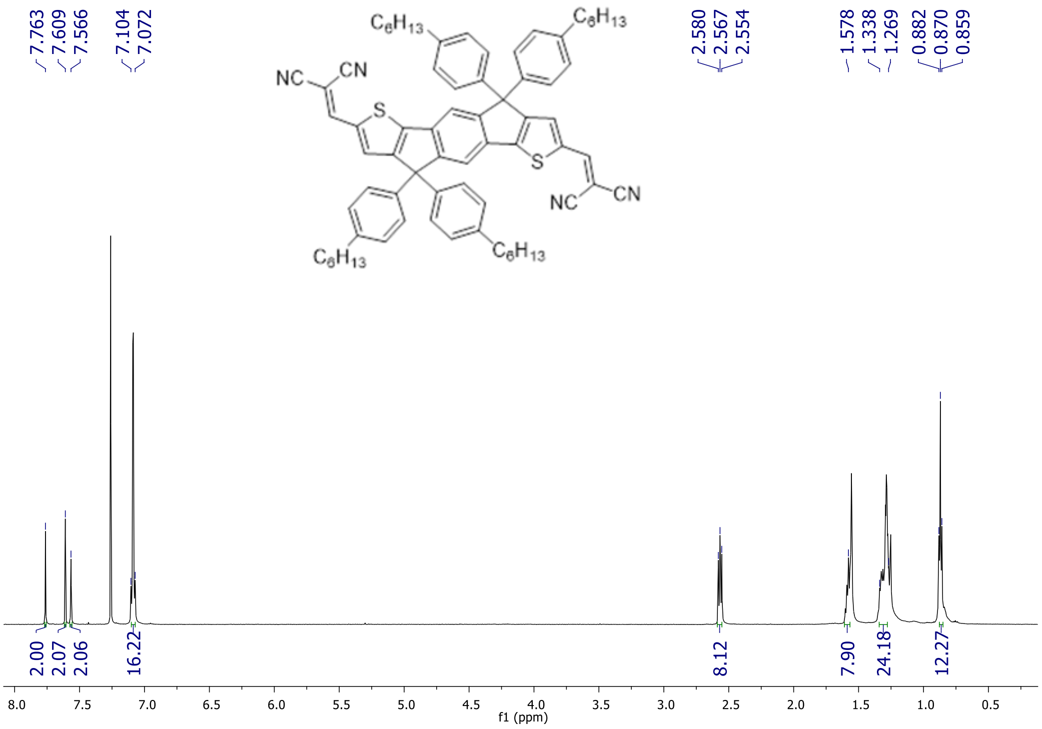

Figure S1. ^{1}H NMR ($CDCl_3$, 600 MHz) spectrum of compound IDT-1

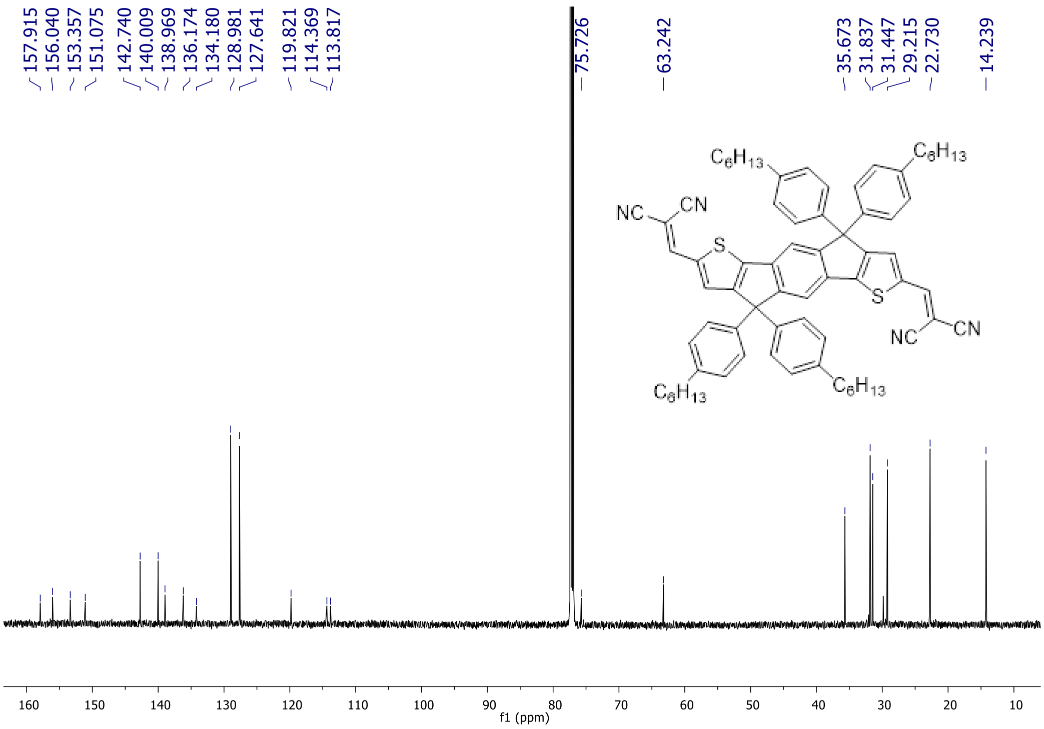

Figure S2. ^{13}C NMR ($CDCl_3$, 150 MHz) spectrum of compound IDT-1

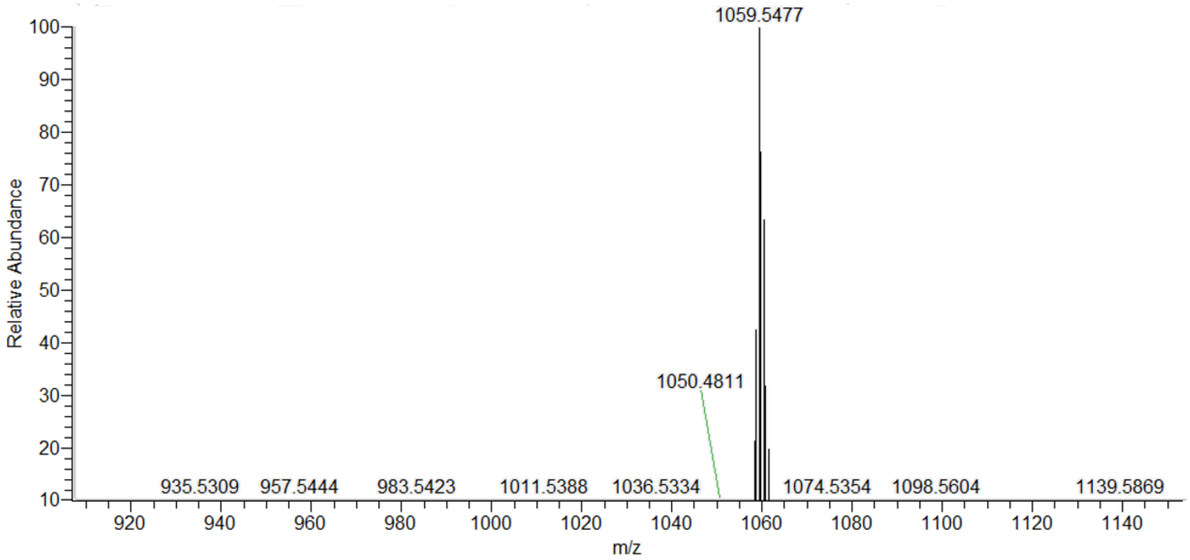

Figure S3. APCI (+)-HRMS spectrum of compound IDT-1

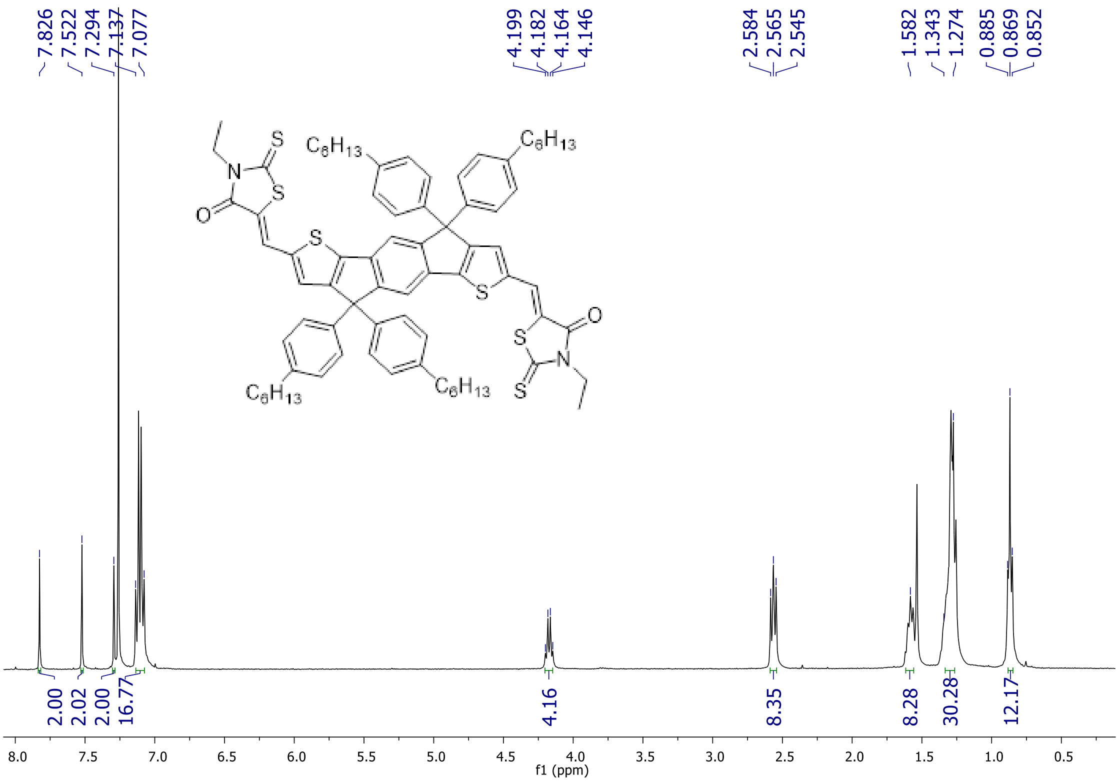

Figure S4. ^{1}H NMR ($CDCl_3$, 400 MHz) spectrum of compound IDT-2

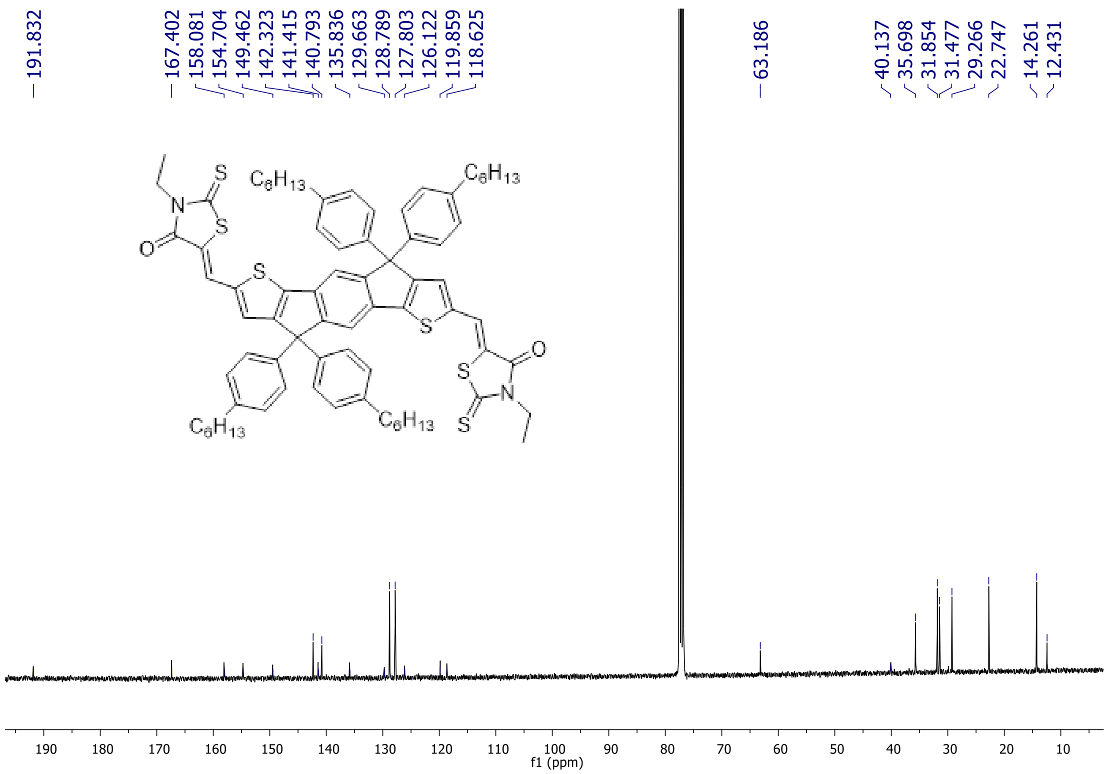

Figure S5. ^{13}C NMR ($CDCl_3$, 100 MHz) spectrum of compound IDT-2

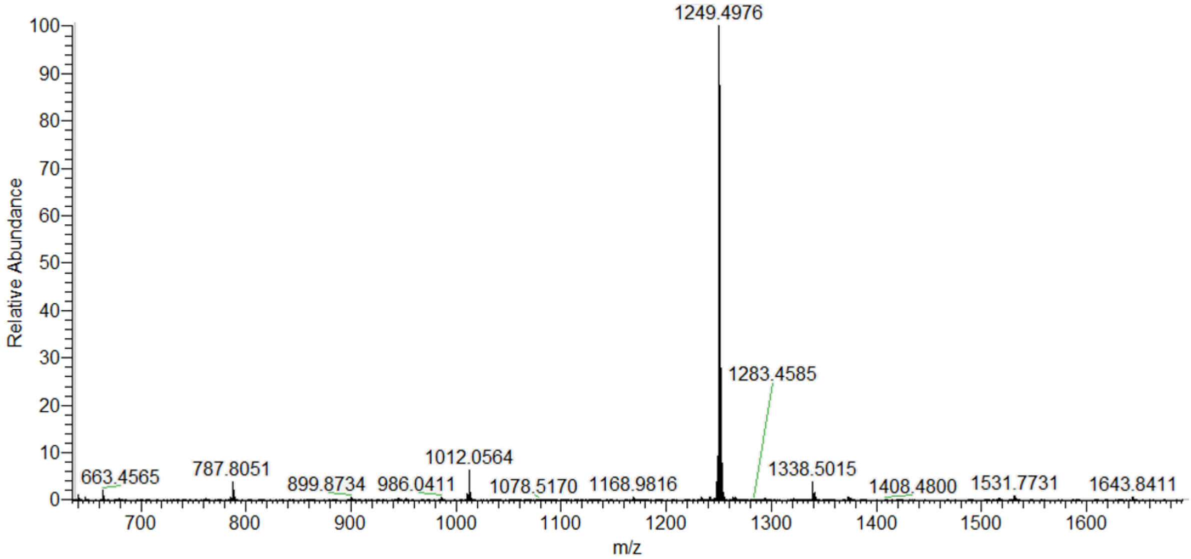

Figure S6. APCI (+)-HRMS spectrum of compound IDT-2

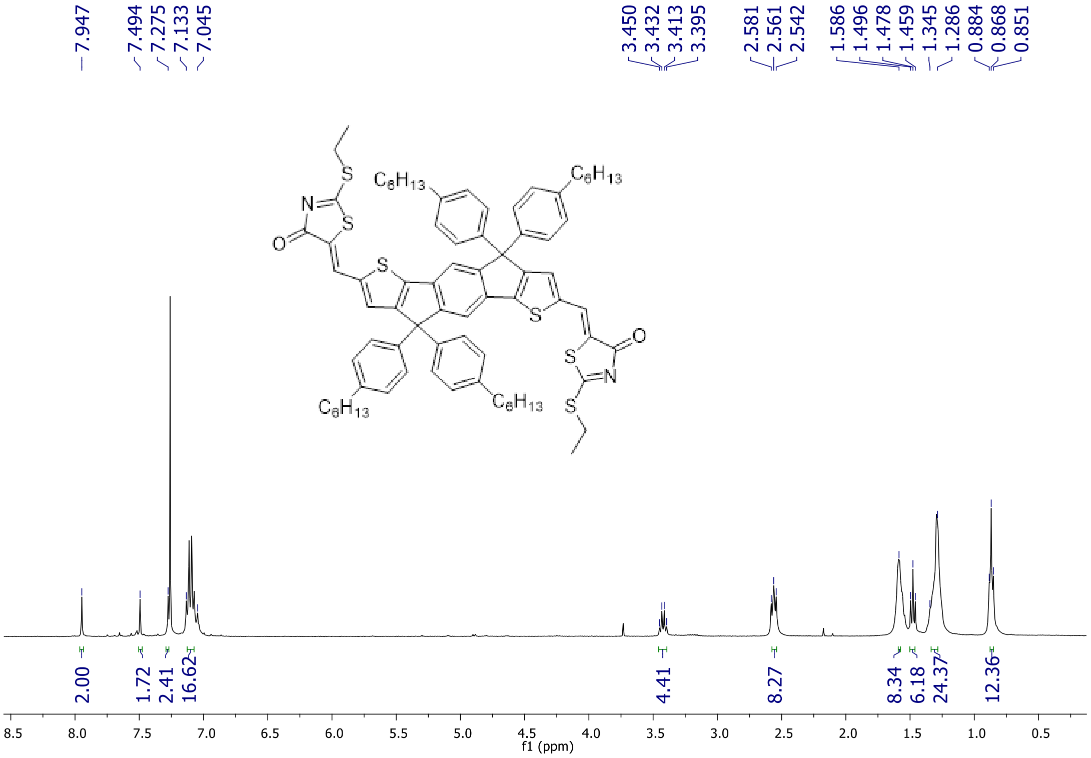

Figure S7. ^{1}H NMR ($CDCl_3$, 400 MHz) spectrum of compound IDT-3

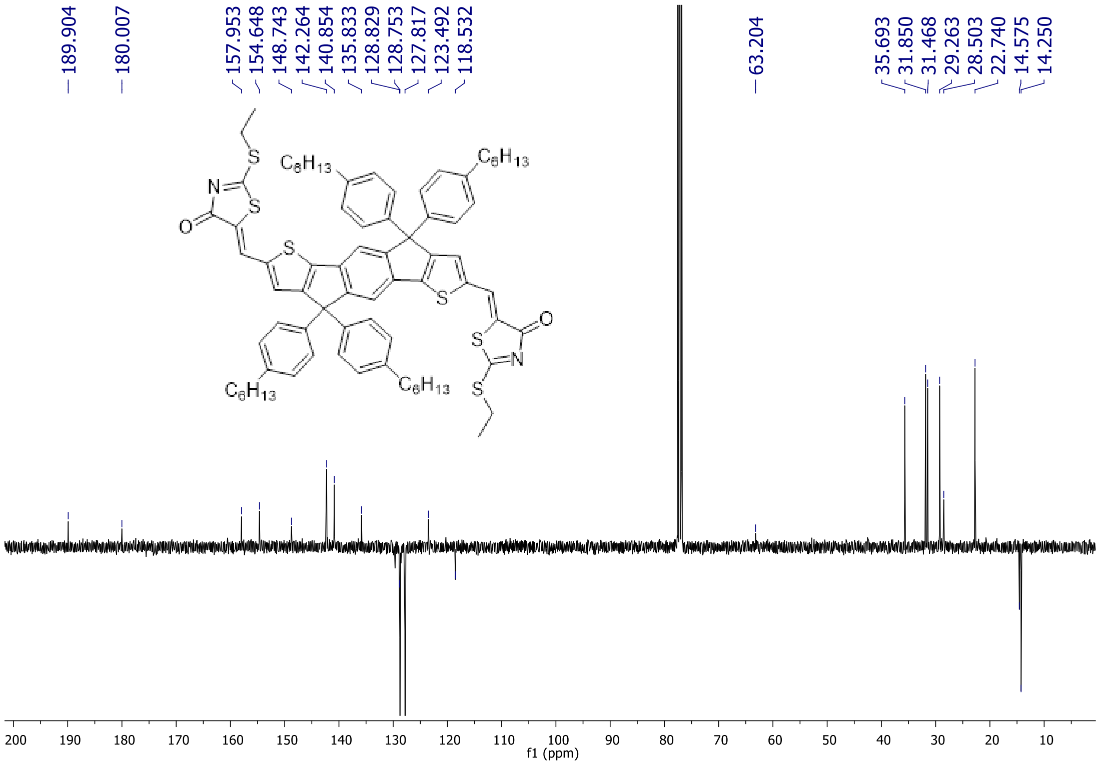

Figure S8. ^{13}C-APT NMR ($CDCl_3$, 100 MHz) spectrum of compound IDT-3

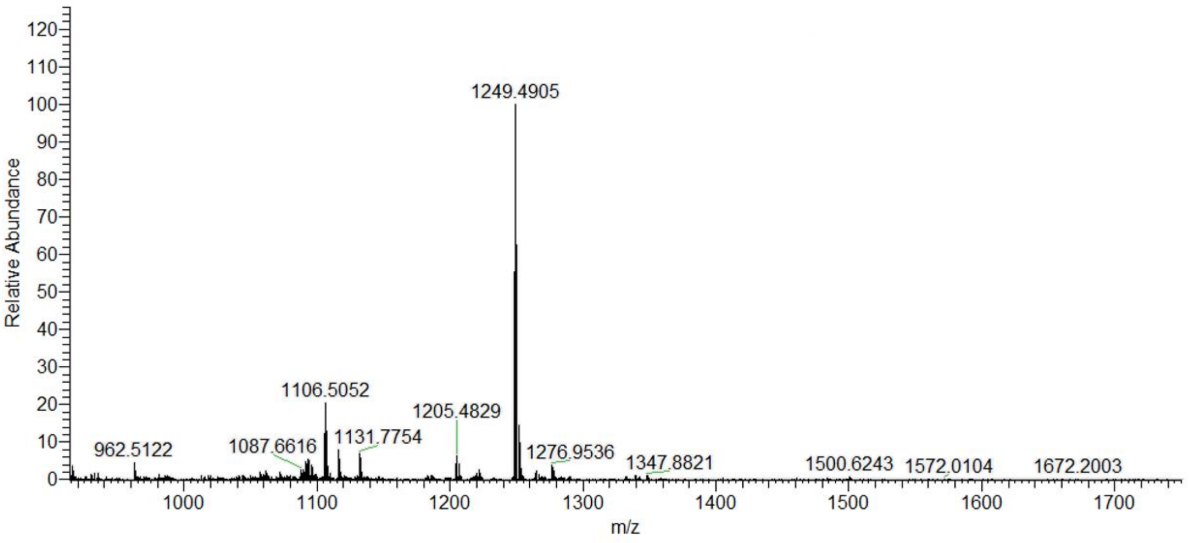

Figure S9. ESI (+)-HRMS spectrum of compound IDT-3

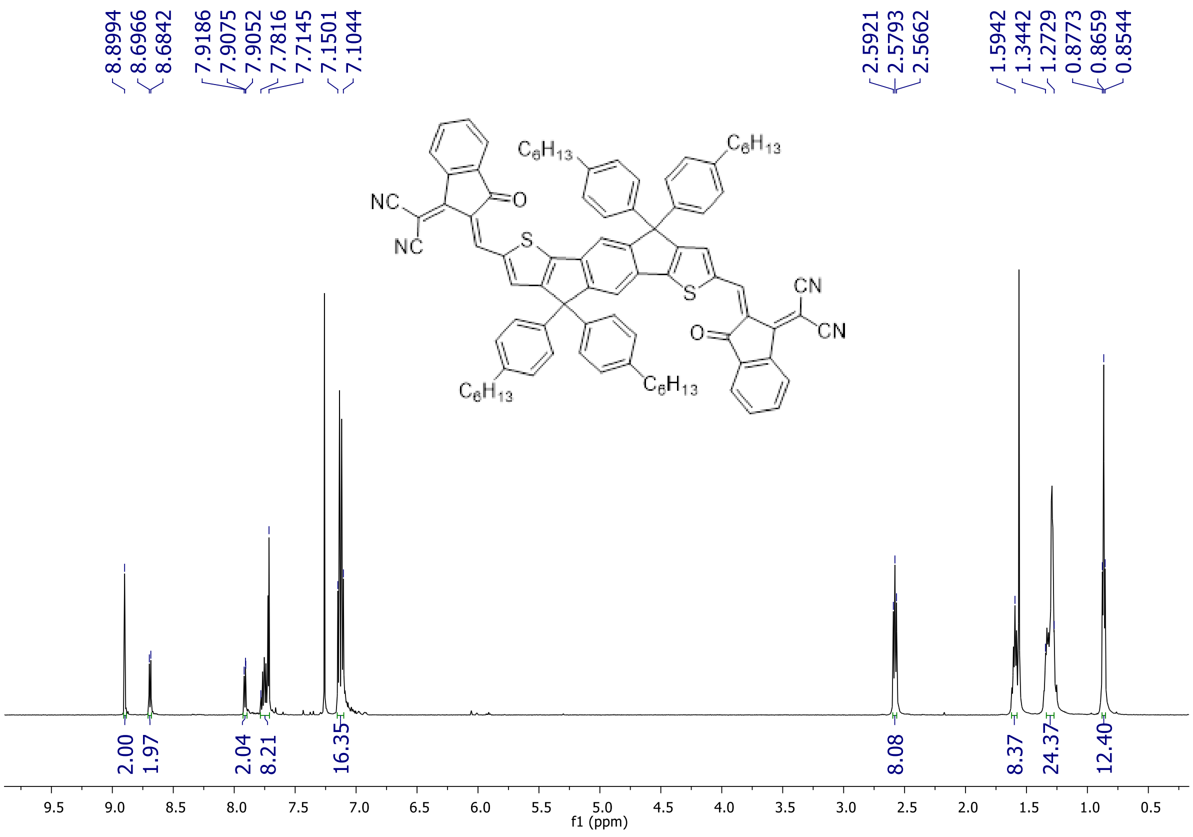

Figure S10. ^{1}H NMR ($CDCl_3$, 600 MHz) spectrum of compound IDT-4

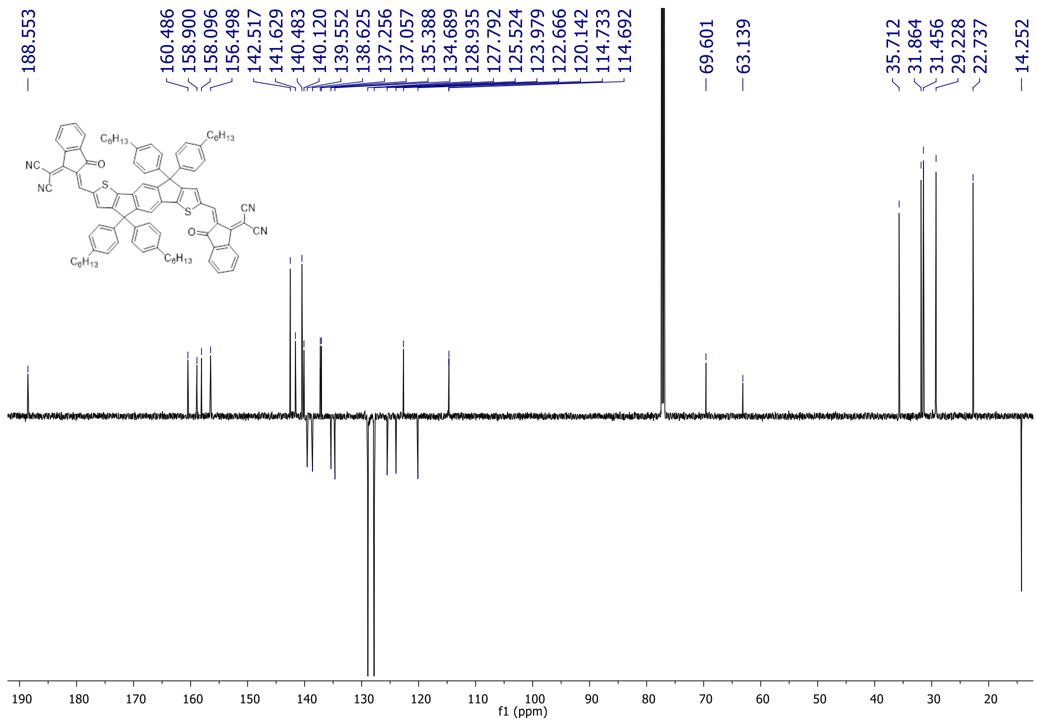

Figure S11. ^{13}C-APT NMR ($CDCl_3$, 150 MHz) spectrum of compound IDT-4

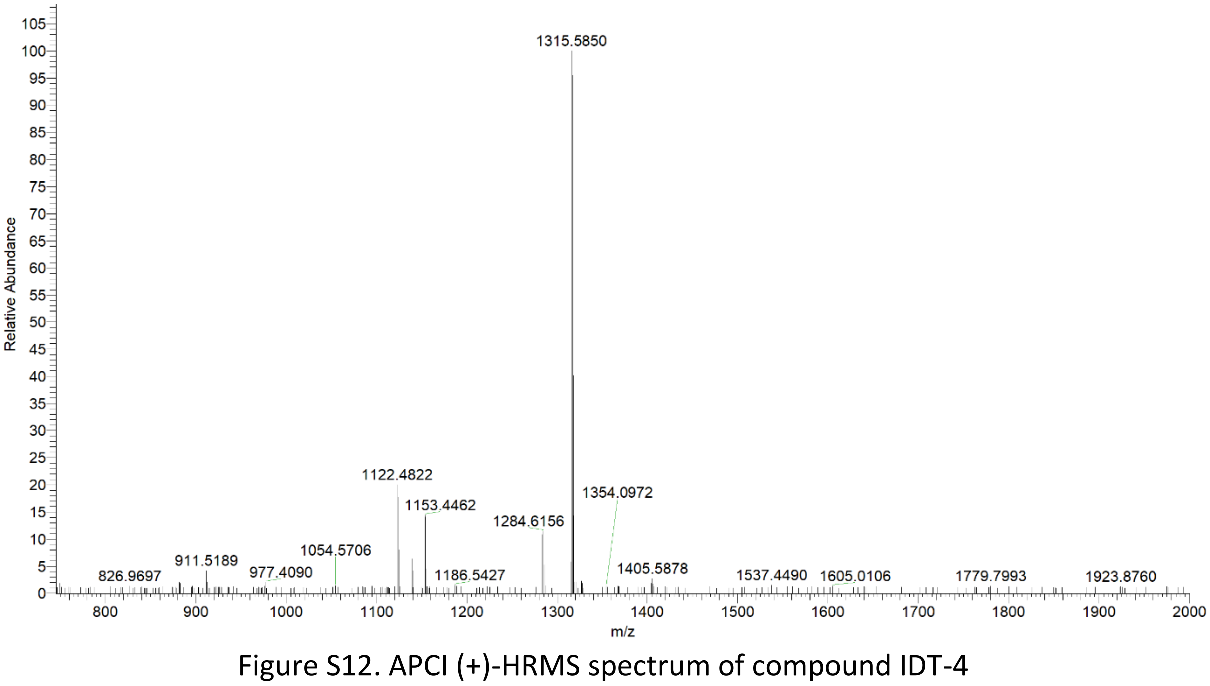

Figure S12. APCI (+)-HRMS spectrum of compound IDT-4

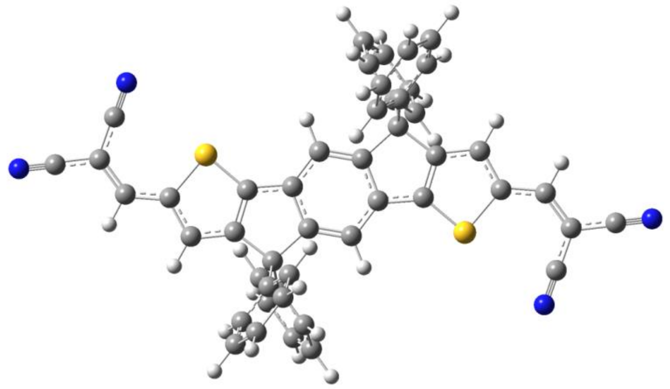

Figure S13. DFT calculated structure of IDT-1

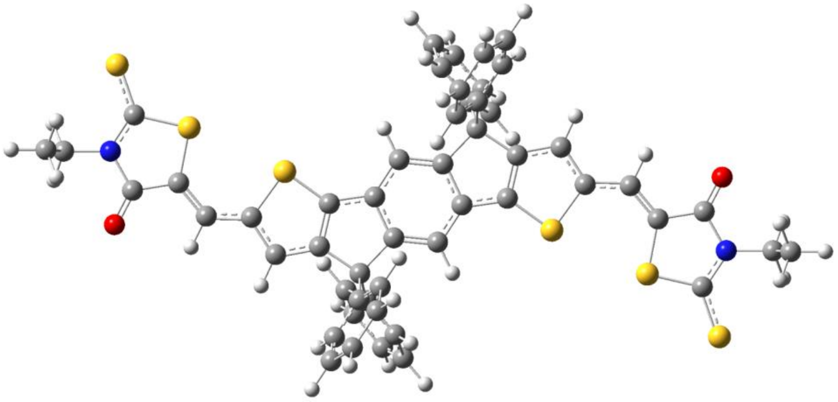

Figure S14. DFT calculated structure of IDT-2

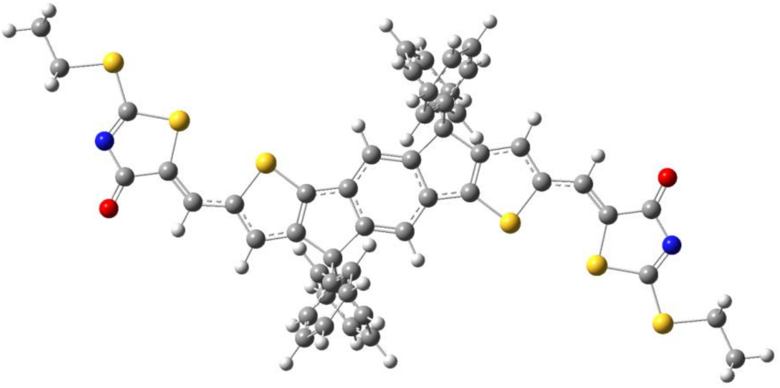

Figure S15. DFT calculated structure of IDT-3

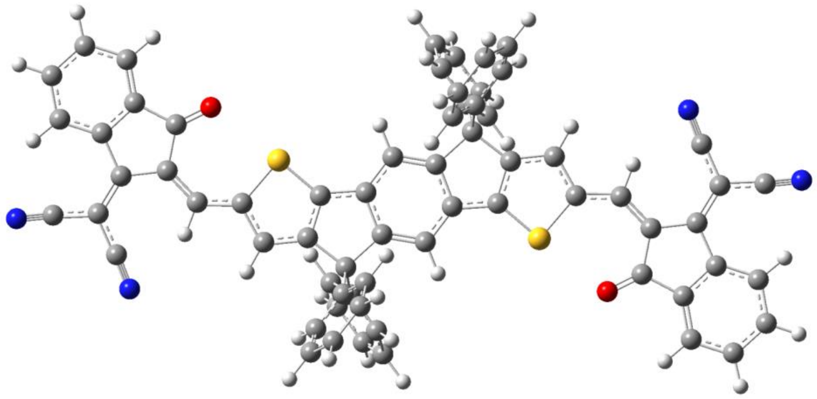

Figure S16. DFT calculated structure of IDT-4

Table S1. DFT coordinates of IDT-1

E=-2860.70124808 Hartrees

E_{LUMO}= -0,12743 Hartrees

E_{HOMO}= -0,22074 Hartrees

C	5.21682800	0.67517700	-0.03636300
C	4.42006100	1.82496400	-0.03438900
C	3.05705300	1.53353000	-0.01839500
C	2.79337000	0.17298800	-0.00068500
S	4.21606000	-0.79314200	-0.00804400
H	4.84699900	2.81856700	-0.04753100
C	1.37270700	-0.07937900	0.02018700
C	0.64698600	-1.27587300	0.01662400
C	0.73181600	1.18569800	0.02825700
C	-0.73195000	-1.18574300	0.02863400
H	1.14909900	-2.23507200	0.00174200
C	-0.64711400	1.27581900	0.01551800
C	-1.37283600	0.07932700	0.01946600
H	-1.14922100	2.23500600	-0.00020500
C	-2.79348800	-0.17305300	-0.00197000
C	-3.05716200	-1.53360600	-0.01900300
S	-4.21617300	0.79307300	-0.01067500
C	-4.42016200	-1.82504700	-0.03559500
C	-5.21692600	-0.67526100	-0.03868400
H	-4.84708900	-2.81865900	-0.04843100
C	1.75943500	2.33420300	0.01401900
C	-1.75956000	-2.33425800	0.01461000

C	-6.63213200	-0.71440300	-0.06400800
H	-7.04163600	-1.71987500	-0.07810100
C	6.63204800	0.71430700	-0.06089600
H	7.04155700	1.71977300	-0.07532300
C	7.56025800	-0.30002200	-0.06977300
C	-7.56033400	0.29992300	-0.07399700
C	-7.23039600	1.68316200	-0.05822700
N	-6.96477900	2.80904500	-0.04527900
C	-8.95019000	-0.01100800	-0.10242000
N	-10.07964200	-0.25876800	-0.12578800
C	7.23031600	-1.68325300	-0.05341500
N	6.96469400	-2.80912900	-0.03998600
C	8.95013000	0.01089500	-0.09757500
N	10.07959400	0.25864400	-0.12043500
C	-1.52587600	-3.17337100	-1.25393000
C	-0.59265200	-4.21672500	-1.24215500
C	-2.16442700	-2.85745200	-2.45635500
C	-0.30884900	-4.92944200	-2.40442700
H	-0.09395800	-4.48291800	-0.31796100
C	-1.88286000	-3.57262900	-3.61955700
H	-2.88437400	-2.04893000	-2.49015400
C	-0.95466300	-4.61119400	-3.59852300
H	0.41499400	-5.73591000	-2.37471800
H	-2.39198300	-3.31579000	-4.54157100
H	-0.73755700	-5.16879900	-4.50226700
C	-1.75702500	-3.17990000	1.30168200
C	-2.49288300	-4.37168800	1.33611500
C	-1.11417800	-2.75614600	2.46680600
C	-2.57593800	-5.12379800	2.50373500
H	-2.99322700	-4.71972300	0.44001700
C	-1.19579900	-3.51077700	3.63778500
H	-0.54561500	-1.83506500	2.46920200
C	-1.92465200	-4.69648000	3.66126100
H	-3.14694100	-6.04530300	2.50855400
H	-0.68530200	-3.16763700	4.53050200
H	-1.98491400	-5.28371800	4.57026200
C	1.52640900	3.17247200	-1.25520400
C	0.59314500	4.21580200	-1.24461700
C	2.16562400	2.85579200	-2.45707600
C	0.30994800	4.92775200	-2.40750600

H	0.09394400	4.48258700	-0.32086700
C	1.88466300	3.57020000	-3.62089600
H	2.88562200	2.04727700	-2.48995600
C	0.95641900	4.60874600	-3.60104500
H	-0.41393700	5.73421600	-2.37871500
H	2.39429500	3.31277700	-4.54246600
H	0.73978500	5.16575400	-4.50527000
C	1.75624300	3.18070000	1.30052400
C	1.11289100	2.75766200	2.46562900
C	2.49201100	4.37255500	1.33451500
C	1.19392800	3.51306200	3.63615300
H	0.54438900	1.83654400	2.46836200
C	2.57448500	5.12543000	2.50168300
H	2.99274100	4.72003900	0.43841800
C	1.92269500	4.69882600	3.65918900
H	0.68304600	3.17047100	4.52886100
H	3.14542800	6.04697400	2.50616300
H	1.98250500	5.28666000	4.56783500

Table S2. DFT coordinates of IDT-2

E= -4653.16839656 Hartrees

E_{LUMO}= -0,11821 Hartrees

E_{HOMO}= -0,20582 Hartrees

C	5.24584000	0.52532100	-0.28166200
C	4.48585800	1.69371000	-0.28387900
C	3.10994000	1.44141300	-0.27011600
C	2.79936600	0.09415600	-0.25002600
S	4.20033600	-0.90968700	-0.25311200
H	4.94218000	2.67415700	-0.29708500
C	1.37114200	-0.11376200	-0.23070400
C	0.60398300	-1.28422800	-0.22846200
C	0.77110500	1.17066600	-0.22837500
C	-0.77159000	-1.14798900	-0.21646100
H	1.07358600	-2.26009900	-0.23743400
C	-0.60445600	1.30677500	-0.24208400
C	-1.37160900	0.13634800	-0.23229500
H	-1.07406100	2.28249700	-0.26132500
C	-2.79982300	-0.07172100	-0.24981900
C	-3.11044800	-1.41910500	-0.25583500
S	-4.20071500	0.93213500	-0.26398900
C	-4.48639700	-1.67141000	-0.26729500

C	-5.24629800	-0.50301400	-0.27789200
H	-4.94285500	-2.65188100	-0.27036400
C	1.83799000	2.28368400	-0.24340800
C	-1.83851200	-2.26109900	-0.22037500
C	-6.66759300	-0.46588300	-0.29856500
H	-7.14722800	-1.44179600	-0.30215500
C	6.66715200	0.48802200	-0.30074800
H	7.14675200	1.46383300	-0.31639200
C	-7.53715200	0.57715200	-0.31521000
C	7.53674300	-0.55511900	-0.30294300
S	-7.20527000	2.31152200	-0.31505900
S	7.20484700	-2.28930600	-0.27959300
C	-8.93136100	2.70825200	-0.34850900
C	-8.98924100	0.34159900	-0.33655100
C	8.93095500	-2.68645100	-0.30544000
C	8.98886100	-0.31985900	-0.32605400
S	-9.46964700	4.27663100	-0.35404400
S	9.46918800	-4.25476800	-0.28901600
O	-9.55588800	-0.73379700	-0.32892800
O	9.55544900	0.75557000	-0.33282900
N	-9.68244900	1.57102100	-0.36892900
N	9.68210900	-1.54961500	-0.34051100
C	-11.15556300	1.57339100	-0.36205000
H	-11.46047800	0.65978700	-0.87059200
H	-11.48207800	2.43076400	-0.94852200
C	11.15522100	-1.55200900	-0.33176100
H	11.48240700	-2.41678400	-0.90689200
H	11.46084200	-0.64497800	-0.85151300
C	-11.71163400	1.62157400	1.05870300
H	-11.38177200	0.75095200	1.62910400
H	-11.38445500	2.52701400	1.57288100
H	-12.80342800	1.61834000	1.02526500
C	11.70961000	-1.58217600	1.09015000
H	12.80144300	-1.57924200	1.05792400
H	11.37902200	-0.70446600	1.64916000
H	11.38194500	-2.48109100	1.61534100
C	1.63689700	3.12631100	-1.51478200
C	0.73644500	4.19831800	-1.51157100
C	2.27263800	2.78852400	-2.71280700
C	0.48120600	4.91643600	-2.67714200

H	0.24099900	4.48197400	-0.59080400
C	2.01979300	3.50882600	-3.87951200
H	2.96828900	1.95875300	-2.73899300
C	1.12357000	4.57528700	-3.86684600
H	-0.21758300	5.74500800	-2.65365600
H	2.52635500	3.23418000	-4.79787200
H	0.92888500	5.13685900	-4.77329500
C	1.85989500	3.13403900	1.03995000
C	2.63622500	4.30004200	1.07292300
C	1.19880000	2.73789700	2.20471800
C	2.74115700	5.05378700	2.23774000
H	3.15236500	4.62576500	0.17737000
C	1.30254300	3.49383500	3.37314000
H	0.59849400	1.83720700	2.20756600
C	2.07163100	4.65396600	3.39474600
H	3.34403900	5.95483900	2.24107000
H	0.77767000	3.17187300	4.26549900
H	2.14916500	5.24237400	4.30174400
C	-1.86051500	-3.09868000	1.07134100
C	-2.63647100	-4.26455700	1.11573000
C	-1.19973300	-2.69081700	2.23224100
C	-2.74144700	-5.00669100	2.28798100
H	-3.15227500	-4.59943400	0.22336400
C	-1.30350900	-3.43512200	3.40810200
H	-0.59960700	-1.79001400	2.22622300
C	-2.07229800	-4.59518800	3.44109500
H	-3.34404700	-5.90785600	2.30013100
H	-0.77887300	-3.10418700	4.29731200
H	-2.14985400	-5.17457700	4.35387900
C	-1.63719800	-3.11641200	-1.48323500
C	-0.73589600	-4.18762900	-1.46937500
C	-2.27354800	-2.79135900	-2.68444800
C	-0.48039300	-4.91735400	-2.62764200
H	-0.23991400	-4.46145700	-0.54592000
C	-2.02045700	-3.52330700	-3.84383400
H	-2.96983100	-1.96242700	-2.71891700
C	-1.12337400	-4.58885700	-3.82058000
H	0.21908400	-5.74507100	-2.59593400
H	-2.52749700	-3.25842000	-4.76479400
H	-0.92848200	-5.15946800	-4.72132200

Table S3. DFT coordinates of IDT-3

E= -4653.11665209 Hartrees

E_{LUMO}= -0,11170 Hartrees

E_{HOMO}= -0,20271 Hartrees

C	5.27341100	0.42431800	-0.26821200
C	4.54308400	1.60949100	-0.27111800
C	3.15937900	1.39103400	-0.26241400
C	2.81551100	0.05332400	-0.24481400
S	4.19302900	-0.98436200	-0.24488900
H	5.02330700	2.57858300	-0.28086400
C	1.38193200	-0.11965100	-0.22873200
C	0.58553800	-1.27037200	-0.22723800
C	0.81354800	1.17871500	-0.22685700
C	-0.78656700	-1.10016100	-0.21576900
H	1.03099600	-2.25766400	-0.23518100
C	-0.55854100	1.34879000	-0.24149200
C	-1.35494200	0.19810500	-0.23173400
H	-1.00398300	2.33594600	-0.26029500
C	-2.78850300	0.02495700	-0.24730300
C	-3.13238000	-1.31286800	-0.25118400
S	-4.16597900	1.06263100	-0.25929000
C	-4.51610400	-1.53134100	-0.25865500
C	-5.24638500	-0.34618200	-0.26885500
H	-4.99641800	-2.50043800	-0.25864900
C	1.90841800	2.26466000	-0.23941400
C	-1.88142600	-2.18621100	-0.21814900
C	-6.66952500	-0.27020100	-0.28552700
H	-7.17521800	-1.23343100	-0.28515500
C	6.69656400	0.34816000	-0.28271300
H	7.20227400	1.31132600	-0.29304200
C	-7.51550800	0.78779200	-0.30221900
C	7.54253600	-0.70996600	-0.28623900
S	-7.13435800	2.51078800	-0.31136100
S	7.16137000	-2.43293800	-0.27494600
C	-8.90259300	2.83037800	-0.33347600
C	-9.00016200	0.59501800	-0.31757800
C	8.92961100	-2.75278100	-0.29218400
C	9.02720700	-0.51739500	-0.30265100
S	-9.39411600	4.49872000	-0.35129600
S	9.42113700	-4.42120800	-0.29027400

O	-9.54894100	-0.49025400	-0.31580000
O	9.57600100	0.56781900	-0.31315500
N	-9.69626200	1.81777500	-0.33466200
N	9.72330400	-1.74027300	-0.30481500
C	1.73183400	3.11151100	-1.51154800
C	0.85587100	4.20370700	-1.51245200
C	2.36556500	2.76005500	-2.70670000
C	0.62213500	4.92752200	-2.67899000
H	0.36275200	4.49825700	-0.59382900
C	2.13431100	3.48593900	-3.87445600
H	3.04289000	1.91517800	-2.72928000
C	1.26204200	4.57212000	-3.86588300
H	-0.05785600	5.77172000	-2.65855600
H	2.63908900	3.20024400	-4.79044700
H	1.08426200	5.13801700	-4.77314100
C	1.94796100	3.11543900	1.04301600
C	2.75419100	4.26098100	1.07748600
C	1.27324800	2.73798600	2.20623400
C	2.87539300	5.01285400	2.24193800
H	3.28171300	4.57174500	0.18325000
C	1.39325000	3.49202000	3.37435100
H	0.64964700	1.85326400	2.20772500
C	2.19236200	4.63169000	3.39737500
H	3.50194000	5.89763700	2.24640200
H	0.85768300	3.18452500	4.26549600
H	2.28285200	5.21853300	4.30421400
C	-1.92196200	-3.02389900	1.07283600
C	-2.72713100	-4.16980900	1.11805300
C	-1.24916200	-2.63380300	2.23300300
C	-2.84926700	-4.90965500	2.29010200
H	-3.25305800	-4.49045100	0.22636700
C	-1.37008400	-3.37578400	3.40870700
H	-0.62632400	-1.74856000	2.22613600
C	-2.16820800	-4.51589000	3.44245800
H	-3.47498100	-5.79494700	2.30286600
H	-0.83600300	-3.05859700	4.29734300
H	-2.25941300	-5.09338300	4.35521000
C	-1.70363000	-3.04594800	-1.48144100
C	-0.82662600	-4.13726700	-1.47072100
C	-2.33724100	-2.70751900	-2.68040100

C	-0.59171400	-4.87287300	-2.62960500
H	-0.33357200	-4.42179900	-0.54890800
C	-2.10480800	-3.44521600	-3.84050200
H	-3.01536900	-1.86358100	-2.71201000
C	-1.23148500	-4.53038400	-3.82037200
H	0.08909300	-5.71614700	-2.60017400
H	-2.60950300	-3.16949100	-4.75959000
H	-1.05277600	-5.10545400	-4.72166000
C	-11.23284800	4.31862800	-0.37481000
H	-11.48805700	3.73527400	-1.25843600
H	-11.51217400	3.74964400	0.51086400
C	-11.87313400	5.70245600	-0.39489000
H	-11.58378600	6.26873800	-1.28305800
H	-12.95959100	5.58798300	-0.40819600
H	-11.60713000	6.28380800	0.49083300
C	11.25991100	-4.24133400	-0.31148300
H	11.53713100	-3.66267200	0.56857900
H	11.51720400	-3.66767800	-1.20082300
C	11.90021400	-5.62529500	-0.31483400
H	11.61285400	-6.20135600	-1.19734200
H	12.98670300	-5.51098400	-0.32694500
H	11.63218100	-6.19681300	0.57666300

Table S4. DFT coordinates of IDT-4

E= -4653.11665209 Hartrees

E_{LUMO}= -0,13405 Hartrees

E_{HOMO}= -0,21535 Hartrees

C	5.24342700	0.46316000	-0.01326200
C	4.48136000	1.64589400	-0.01099900
C	3.11252900	1.41346800	-0.00282000
C	2.79932800	0.06043300	0.00963400
S	4.17923000	-0.96578400	0.00519700
H	4.94799000	2.62190800	-0.01738300
C	1.36977000	-0.13181300	0.02643200
C	0.59743300	-1.29976900	0.02209200
C	0.77668200	1.15726700	0.03541400
C	-0.77670100	-1.15727300	0.03484300
H	1.06376200	-2.27701700	0.00799900
C	-0.59744200	1.29976100	0.02154300
C	-1.36978200	0.13180500	0.02530700
H	-1.06375900	2.27700800	0.00701800

C	-2.79932600	-0.06044200	0.00734700
C	-3.11251600	-1.41347900	-0.00528600
S	-4.17922100	0.96577700	0.00172700
C	-4.48134200	-1.64590300	-0.01457700
C	-5.24340400	-0.46316800	-0.01750800
H	-4.94797100	-2.62191500	-0.02128500
C	1.84802900	2.26517400	0.02722200
C	-1.84803900	-2.26518100	0.02587100
C	-6.65765700	-0.52844600	-0.03626400
H	-6.97588100	-1.56240400	-0.04536600
C	6.65769400	0.52843700	-0.03096600
H	6.97592600	1.56239300	-0.03986200
C	-7.66586300	0.40869800	-0.04342600
C	-9.79581700	1.45652200	-0.04844200
C	-8.83020500	2.47627200	-0.02589700
C	-11.14862000	1.80635500	-0.05226100
C	-9.16240200	3.82102900	-0.00823600
C	-11.48961100	3.16043600	-0.03409100
H	-11.93454700	1.06814800	-0.06813200
C	-10.51437900	4.16206900	-0.01259900
H	-8.38252600	4.57267800	0.00860000
H	-12.53720800	3.43684100	-0.03664900
H	-10.81322800	5.20327500	0.00092100
C	7.66590300	-0.40870900	-0.03736600
C	9.79586100	-1.45653000	-0.04063400
C	8.83023400	-2.47627900	-0.01867200
C	11.14866800	-1.80636100	-0.04336700
C	9.16242100	-3.82103200	-0.00056100
C	11.48964700	-3.16043800	-0.02473600
H	11.93460400	-1.06815400	-0.05874200
C	10.51440100	-4.16207000	-0.00384400
H	8.38253400	-4.57268100	0.01579000
H	12.53724700	-3.43684000	-0.02645100
H	10.81324200	-5.20327400	0.01005500
C	-9.10480000	0.14211800	-0.06480600
C	-7.48070800	1.87627600	-0.02351500
O	-6.42505300	2.49020300	-0.00660300
C	9.10485400	-0.14213100	-0.05776300
C	7.48073400	-1.87628500	-0.01740800
O	6.42506800	-2.49021200	-0.00121300

C	9.74790300	1.08117500	-0.09208900
C	-9.74783000	-1.08119400	-0.09927300
C	-9.08754400	-2.34303200	-0.12408500
N	-8.60267000	-3.39362700	-0.14628400
C	-11.16404700	-1.23004900	-0.11761300
N	-12.30628100	-1.41304900	-0.13370200
C	9.08762900	2.34300000	-0.11779000
N	8.60276300	3.39358400	-0.14069200
C	11.16413400	1.23003200	-0.10940700
N	12.30637800	1.41303300	-0.12468200
C	-1.65128800	-3.11690600	-1.24037300
C	-0.75500700	-4.19225000	-1.22927300
C	-2.28641300	-2.78529000	-2.44030400
C	-0.50335300	-4.92001300	-2.38956500
H	-0.26054200	-4.47128400	-0.30656900
C	-2.03739400	-3.51557500	-3.60157500
H	-2.97895700	-1.95319800	-2.47304900
C	-1.14533200	-4.58533300	-3.58128300
H	0.19198300	-5.75125100	-2.36021200
H	-2.54389500	-3.24621700	-4.52148000
H	-0.95378600	-5.15470200	-4.48350200
C	-1.87507000	-3.10840800	1.31396700
C	-2.66411400	-4.26559600	1.35335100
C	-1.20721000	-2.71378900	2.47515000
C	-2.77523200	-5.01236400	2.52194400
H	-3.18578700	-4.58970600	0.46042700
C	-1.31658300	-3.46343500	3.64721200
H	-0.59765400	-1.81929900	2.47365700
C	-2.09871100	-4.61455100	3.67565100
H	-3.38867800	-5.90614700	2.53082200
H	-0.78634800	-3.14318700	4.53697700
H	-2.18127200	-5.19763500	4.58561600
C	1.87401300	3.10853500	1.31524900
C	2.66301400	4.26573600	1.35514600
C	1.20522500	2.71402700	2.47593600
C	2.77318600	5.01262600	2.52375100
H	3.18540500	4.58975700	0.46261000
C	1.31365100	3.46379500	3.64800700
H	0.59568400	1.81952700	2.47404200
C	2.09574300	4.61492300	3.67695500

H	3.38661400	5.90641600	2.53302900
H	0.78270700	3.14363300	4.53738100
H	2.17756700	5.19810200	4.58692600
C	1.65231600	3.11677400	-1.23926900
C	0.75594600	4.19205400	-1.22902700
C	2.28849700	2.78511000	-2.43862700
C	0.50523400	4.91970500	-2.38959200
H	0.26066200	4.47112300	-0.30677300
C	2.04042100	3.51528300	-3.60017000
H	2.98113100	1.95306800	-2.47071100
C	1.14826400	4.58497700	-3.58073200
H	-0.19018900	5.75089300	-2.36090400
H	2.54773200	3.24588900	-4.51961800
H	0.95745000	5.15425900	-4.48316100

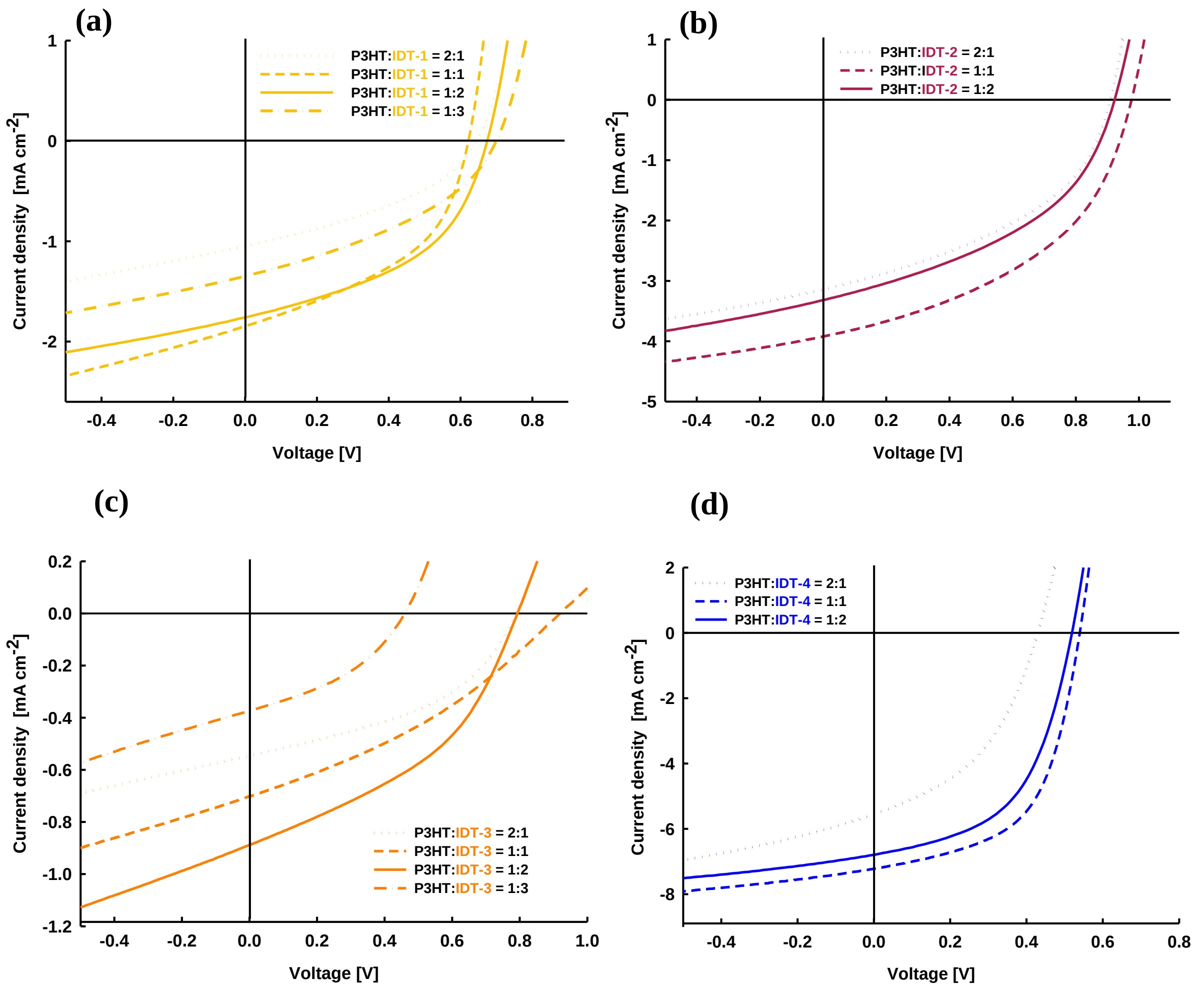

Figure S17. *J-V* characteristics of BHJ OSCs for different P3HT : *n*-type molecule ratios. (a) IDT-1, (b) IDT-2, (c) IDT-3 and (d) IDT-4

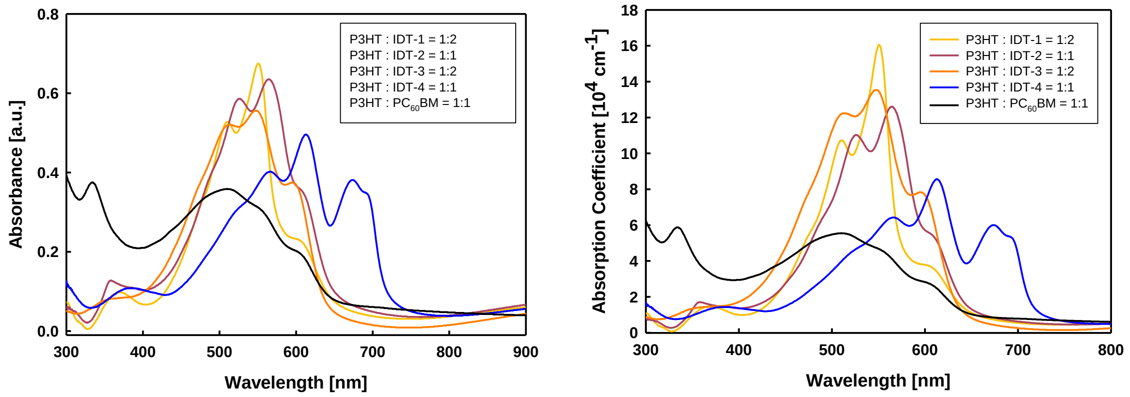

Figure S18. Absorption spectra of P3HT : *n*-type molecule blend films of *ca* 70-80 nm thickness

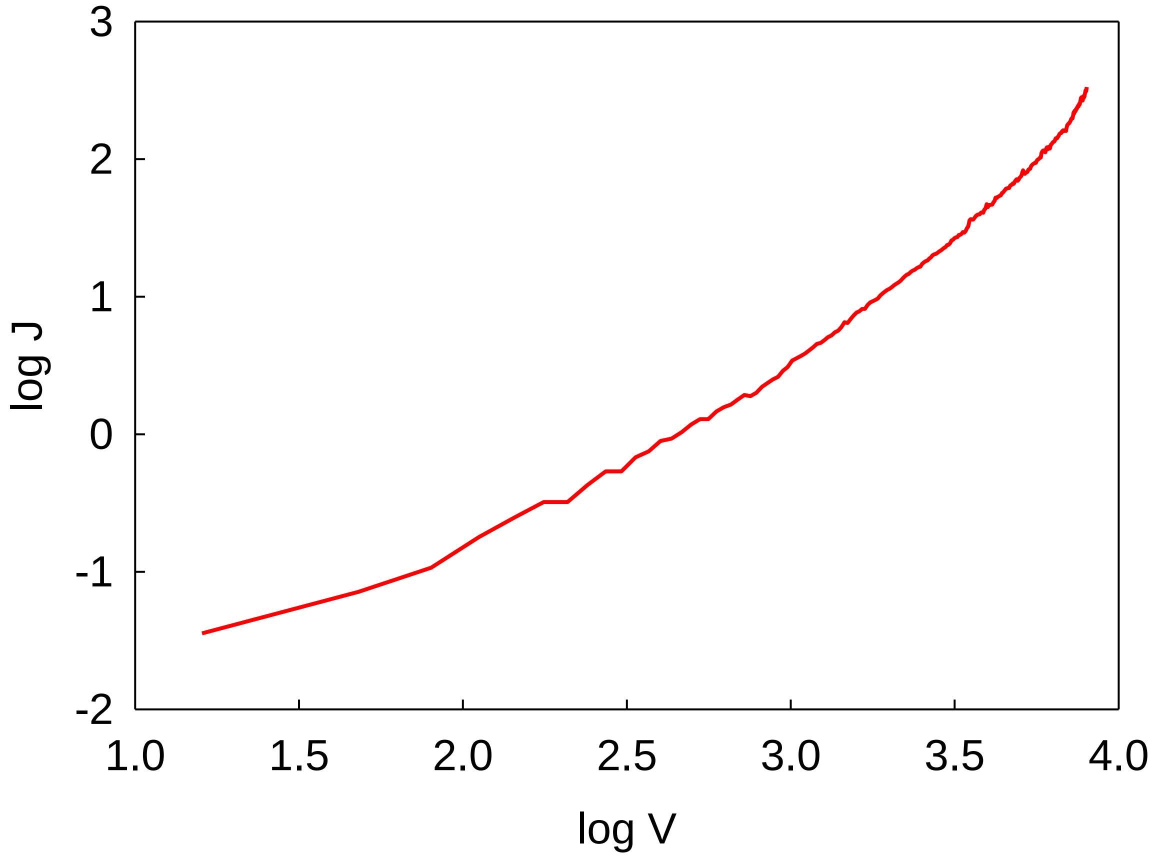

Figure S19. Log J-log V curve of the electron-only devices with the structure of ITO/ZnO (20nm)/**IDT-2**(50nm)/Bphen (15 nm)/Ag (100 nm)

Data extracted from Figure S19 that are used to calculate the electron mobility

**Log(V[mV])**	**Log(J [mA/cm^2])**	**V [V]**	**J [A/cm^2]**	**L [cm]**	**μ_e [cm^2/Vs]**
2.89	0.2616	0.7762	0.001826417	0.000005	1.26927E-06

Table S5. Photovoltaic characteristics of IDT-1, IDT-2, IDT-3, IDT-4 and $PC_{60}BM$ under various conditions

	**D:A**	**rpm/s**	**Thickness [nm]**	**V_{oc} [V]**	**J_{sc} [$mAcm^{-2}$]**	**FF [%]**	**PCE[a] [%]**
IDT-1	1:1	1000/40 + 8000/20	70	0.62	1.85	45.00	0.52 (0.37)
	1:2		70	0.67	1.76	46.32	0.55 (0.52)
	1:3		70	0.70	1.35	38.34	0.36 (0.31)
	2:1		70	0.64	1.05	38.36	0.26 (0.19)
IDT-2	1:1	1000/40 + 8000/20	80	0.97	3.91	45.56	1.74 (1.67)
	1:2		80	0.92	3.32	43.54	1.33 (1.28)
	2:1		80	0.91	3.14	43.12	1.23 (1.14)
IDT-3	1:1	1000/40 + 8000/20	60	0.82	0.82	38.47	0.26 (0.22)
	1:2		60	0.79	0.89	41.31	0.29 (0.26)
	1:3		60	0.46	0.37	38.84	0.07 (0.06)
	2:1		60	0.79	0.55	43.28	0.19 (0.16)
IDT-4	1:1	1000/40 + 8000/20	80	0.54	7.23	56.53	2.21 (2.16)
	1:2		80	0.52	6.78	52.51	1.85 (1.66)
	2:1		80	0.43	5.55	43.11	1.03 (0.92)
$PC_{60}BM$	1:1	1500/40+ 3000/20	80	0.53	3.32	17.45	0.34 (0.26)
	1:1	1500/40+ 3000/20	80	0.51[b]	6.44[b]	52.98[b]	1.72 (1.51)[b]
	1:1	1500/60+ 4000/20	150	0.54	6.29	52.53	1.77 (1.69)
	1:1	1000/60 +3000/20	200	0.59	8.26	56.32	2.76 (2.43)
	1:0.8	1000/60 +3000/20	170	0.58	8.00	53.95	2.50 (2.43)
	1:0.6	500/60 +3000/20	250	0.52	6.36	51.40	1.70 (1.60)

[a] *Average power conversion efficiencies are given in parenthesis.*

[b] *After 10 min thermal treatment at 130 °C.*

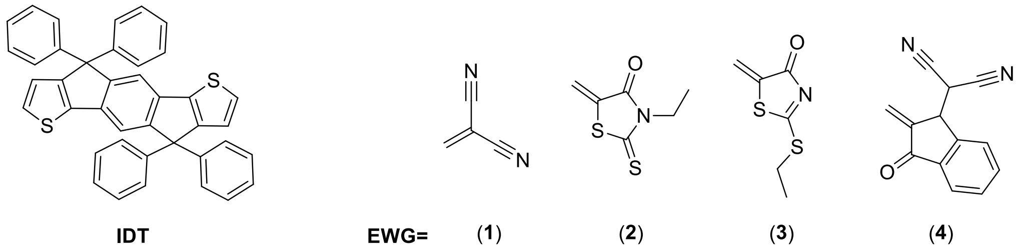

	E_{HOMO} (eV)	E_{LUMO} (eV)
**IDT**	-5.45	-1.71
**EWG=(1)**	-8.50	-2.74
**EWG=(2)**	-6.75	-2.69
**EWG=(3)**	-6.89	-2.45
**EWG=(4)**	-7.36	-3.46

Chart S1. HOMO-LUMO energy levels for the IDT core and electron-withdrawing groups
